# Exonic splicing code and protein binding sites for calcium

**DOI:** 10.1093/nar/gkac270

**Published:** 2022-04-26

**Authors:** Reuben J Pengelly, Dara Bakhtiar, Ivana Borovská, Jana Královičová, Igor Vořechovský

**Affiliations:** University of Southampton, Faculty of Medicine, Southampton SO16 6YD, UK; University of Southampton, Faculty of Medicine, Southampton SO16 6YD, UK; Slovak Academy of Sciences, Centre of Biosciences, 840 05 Bratislava, Slovak Republic; University of Southampton, Faculty of Medicine, Southampton SO16 6YD, UK; Slovak Academy of Sciences, Centre of Biosciences, 840 05 Bratislava, Slovak Republic; Slovak Academy of Sciences, Institute of Zoology, 845 06 Bratislava, Slovak Republic; University of Southampton, Faculty of Medicine, Southampton SO16 6YD, UK

## Abstract

Auxilliary splicing sequences in exons, known as enhancers (ESEs) and silencers (ESSs), have been subject to strong selection pressures at the RNA and protein level. The protein component of this splicing code is substantial, recently estimated at ∼50% of the total information within ESEs, but remains poorly understood. The ESE/ESS profiles were previously associated with the Irving-Williams (I-W) stability series for divalent metals, suggesting that the ESE/ESS evolution was shaped by metal binding sites. Here, we have examined splicing activities of exonic sequences that encode protein binding sites for Ca^2+^, a weak binder in the I-W affinity order. We found that predicted exon inclusion levels for the EF-hand motifs and for Ca^2+^-binding residues in nonEF-hand proteins were higher than for average exons. For canonical EF-hands, the increase was centred on the EF-hand chelation loop and, in particular, on Ca^2+^-coordinating residues, with a 1>12>3∼5>9 hierarchy in the 12-codon loop consensus and usage bias at codons 1 and 12. The same hierarchy but a lower increase was observed for noncanonical EF-hands, except for S100 proteins. EF-hand loops preferentially accumulated exon splits in two clusters, one located in their N-terminal halves and the other around codon 12. Using splicing assays and published crosslinking and immunoprecipitation data, we identify candidate trans-acting factors that preferentially bind conserved GA-rich motifs encoding negatively charged amino acids in the loops. Together, these data provide evidence for the high capacity of codons for Ca^2+^-coordinating residues to be retained in mature transcripts, facilitating their exon-level expansion during eukaryotic evolution.

## INTRODUCTION

Splicing of messenger RNA precursors (pre-mRNAs) removes introns and joins exons together to generate mRNA for translation (1). This process is assisted by evolutionarily conserved but degenerate signals in the pre-mRNA that are recognized by a large RNA-protein complex assembled *ad hoc* on each intron, known as the spliceosome (2). Apart from traditional motifs (splice sites, lariat-intron branch points and polypyrimidine tracts), these signals include exonic splicing enhancers (ESEs) and silencers (ESSs) as well as additional intronic regulatory elements (1). ESEs and ESSs are short pre-mRNA motifs enriched in exons and depleted in introns that promote and inhibit inclusion of coding segments in mRNAs, respectively (3–5). The best characterized ESE/ESS sets have been derived for hexamers (3–6), which represent common and often minimal binding platforms for RNA-binding proteins (RBPs); in addition, mutation-induced splicing defects can be frequently rescued by compensatory mutations within the hexamer radius (7,8). ESEs and ESSs are important both for constitutive and alternative splicing (AS) (4–6,8) and evolved by accommodating interweaved information from the splicing and protein codes (4,6,9–12). The splicing code involves combinations of hundreds of RNA features that predict tissue-dependent changes in splicing patterns, including the intrinsic strength of splice sites, RNA secondary structure and binding sites for RBPs (10). In contrast to the extensively studied splicing code, the protein component that governed ESE/ESS evolution remains largely unexplored. Our limited insight stems from difficulties to uncouple sequence requirements imposed by evolutionary protein-coding pressures from those necessary for correct splicing, even if the two constraints can be partially separated at wobble codon positions (8,9,13).

We have recently proposed that the ESE/ESS evolution had been substantially shaped by codons for amino acids critically contributing to protein binding sites for biologically available divalent metals (14). We noticed that the ESE/ESS profiles for codons important for formation of protein binding sites for divalent metals mirrored the natural order of their affinities, known as the Irving-Williams (I-W) stability series (Ca^2+^≈Mg^2+^<Mn^2+^<Fe^2+^<Cu^2+^≥ Zn^2+^) (15). We found that the weakest binders in the series (Ca^2+^, Mg^2+^) preferentially contacted residues encoded by splicing-enhancing codons whereas codons for residues interacting with tight binders (Cu^2+^, Zn^2+^) usually acted as splicing repressors, with moderate binders (Mn^2+^, Fe^2+^) exhibiting intermediate values of predicted splicing activities (14). This splicing dichotomy suggested that binding sites for divalent metals influenced ESE/ESS evolution by promoting sites for weak metals and repressing sites for highly competitive binders at the exon level. Mutations leading to a loss of codons critical for binding sites for weak metals (or a gain of codons required for sites binding competitive metals) have the opposite, exon-repressing effects, which was illustrated by an ESE-to-ESS conversion at a site required for Ca^2+^-controlled enzymatic activation (14,16). Because ESEs are conserved in vertebrates (17), the splicing-enhancing activity of codons important for binding sites for weak metals may have contributed to their extraordinary expansion during eukaryotic evolution, particularly sites bound by Ca^2+^ (14,18). The expansion of Ca^2+^-binding sites (CBSs) was not entirely matched by binding sites for more competitive metals in the I-W affinity order (18), which require mainly exon-repressive codons for His and Cys (14). The link between ESEs/ESSs and the I-W stability series also suggested that ESE/ESS evolution may have been constrained by chemical properties of divalent metal ions, such as decreasing ionic radius and increasing electron affinity, polarization and covalence, and that these motifs may have evolved to facilitate matching a correct metal ion to its cognate protein binding site (14). However, these hypotheses have not been tested using *bona fide* exonic sequences and cellular mechanisms underlying the relentless evolutionary expansion of CBSs remain poorly understood.

In the present study, we begin to address these questions by analyzing the splicing potential of exons that encode CBSs and their flanking intronic sequences. We also characterize the intron-exon organization of the most prevalent family of Ca^2+^-binding proteins (CBPs) and identify candidate trans-acting factors that preferentially bind to exonic segments coding for the EF-hand Ca^2+^-binding loops. Finally, we discuss how the ESE/ESS evolution may have facilitated signaling of this allosteric metal *par excellence* and a pivotal intracellular second messenger in eukaryotes (19,20).

## MATERIALS AND METHODS

### Extraction and validation of exons that code for Ca^2+^-binding sites

A non-redundant set of human CBSs (Dataset S1) was compiled using the following resources: (i) UniprotID (21); (ii) Protein Data Bank (PDB, https://www.rcsb.org) and (iii) the Database of metal binding sites (MetalDB) (http://metalweb.cerm.unifi.it/), which contain information on metal-binding sites detected in three-dimensional structures (22). We included CBSs with both structural (X-ray crystallography/NMR) (22) and PROSITE-inferred (23) evidence for Ca^2+^ binding. Because ESEs/ESSs in mammals and other vertebrates are conserved (17), we included human sequences that were orthologous to solved mammalian structures, both with and without evidence for metal identity in the crystal. Rare structures solved using yttrium and ytterbium (24,25) were also included. All retrieved sequences were cross-checked against UniprotID (21) and matched to nucleotide (nt) sequences of Ensembl (v. 104) transcripts (26). These selection criteria were adopted to achieve the right balance between accuracy of the dataset, the strength of evidence for Ca^2+^ binding and a sufficient sample size.

CBSs were categorized into four groups: (i) canonical EF-hands (*n* = 296), (ii) noncanonical EF-hands, excluding pseudoEF-hands of S100 proteins (*n* = 66), (iii) S100 proteins (*n* = 15) and (iv) nonEF-hands (*n* = 555) (Dataset S1). Classification into canonical and noncanonical EF-hand proteins was greatly aided by the previous work (27,28) and was confirmed by the ScanProsite sequence analyzer tool (https://prosite.expasy.org/scanprosite/). The tool detects PROSITE signature matches (29), including the 12 amino-acid EF-hand consensus D-(W)-[DNS]-(ILVFYW)-[DENSTG]-[DNQGHRK]-(GP)-[LIVMC]-[DENQSTAGC]-x-x-[DE], where squared brackets surround permitted residues and parentheses residues not allowed. CBSs were encoded by a total of 330 different genes, including 167 genes for canonical EF-hands, 52 for noncanonical EF-hands, 15 for S100 proteins and 158 genes CBPs containing nonEF-hand motifs. We also extracted 60-nt intronic sequences (Ensembl; v. 104) that flanked exons encoding CBSs in each group to characterize their exon-intron structure and traditional splicing motifs.

### Analysis of auxilliary splicing elements in exons that encode Ca^2+^-binding sites

The choice to include or exclude a pre-mRNA sequence in or from mature transcripts is strongly influenced by the ESE/ESS balance, which exhibits a gradient in exon definition on a continuous scale of exon inclusion capacity (6, 30). We therefore employed a comprehensive set of previously derived ESE and ESS hexamers that were derived by splicing enhancement or silencing *ex vivo* afforded by 4096 hexamers placed at five different positions into two model exons (6). Their ESE/ESSseq scores give reliable estimates of exon inclusion activities and were obtained independently of protein binding affinities to any metals (6). In addition to ESE/ESSseq scores, we calculated frequency ratios for a total of 4728 ESE codons and 4360 ESS codons of the standard genetic code, shown here as ln(ESEf/ESSf). We also determined codon counts in 1182 high-confidence ESEs and 1090 ESSs (6) to compute the ESEc/ESSc ratios. The latter measures provide reasonable estimates of codon-specific splicing activities, perhaps barring stop codons since these translation termination signals might induce capricious non-sense mediated mRNA decay in transient transfections (31), although this possibility was considered unlikely (6). The codon-specific measures were previously related to residue frequencies in binding sites for divalent metals of the I-W affinity series (14) and may help to assess the inclusion capacity of exonic segments for conserved protein domains. As a control exon dataset, we extracted RefSeq sequences of human protein-coding exons as defined by the UCSC Table Browser (https://genome.ucsc.edu/cgi-bin/hgTables, data released on 17 May 2021), comprising ∼35 million hexamers in 218 253 coding segments. We computed mean ESE/ESSseq, ESEf/ESSf and ESEc/ESSc values for control exons devoid of the first nt and the last three nts since these exon positions shape 3′ and 5′ splice sites (3′ or 5′ss) consensus, respectively.

### Reporter and expression constructs

Cloning of the *OGDH* splicing reporter containing mutually exclusive exons 4a and 4b was described previously (14). Exon 4b encodes a DADLD site that is essential for activation of 2-oxoglutarate dehydrogenase (OGDH) by Ca^2+^ (32). The site bears similarities to N-terminal parts of EF-hand loops as it contains a DxDxD motif and hydrophobic residues at loop positions −4, −1 and 8, but has no Gly residue at position 6 or acidic residue at position 12 (32). The reporter may thus be useful as a generic model for studying splicing requirements of exons encoding CBSs, which often contain alternate Asp residues, such as DCxDxSDE motifs in low-density lipoprotein receptors, DxDE motifs in epidermal growth factor-like modules or more widespread Dx[DN]xDG motifs in nonEF-hand structural contexts (33,34 and refs. therein). Apart from the wild-type (WT) *OGDH* reporter, we employed mutated minigenes to examine how ESE-to-ESS conversions alter exon 4b inclusion by candidate RBPs. In mutants, each Asp (D) codon (GAY, where Y is pyrimidine) at DADLD was individually replaced by an exon-silencing CAC codon for His, with each substitution significantly reducing exon 4b inclusion in the mRNA (Table S1) (14). Asp substitutions at the DADLD site resulted in loss of Ca^2+^ sensitivity of OGDH (32) and were shown to diminish Ca^2+^ binding in numerous DxDxD EF-hands (35). Finally, constructs expressing human serine/arginine-rich (SR) proteins were prepared using PCR primers shown in Table S1. PCR products were digested by BamHI/XhoI or HindIII/XhoI and inserted into pcDNA3.1/c-*myc*-HisA (Invitrogen) in frame with C-terminal *myc* tags.

### Transfection experiments

Candidate RBPs for binding to codons for CBSs and Ca^2+^-coordinating residues were compiled from previous studies of their RNA binding preferences (Table S2). Small interfering RNAs (siRNAs) used to downregulate most SR proteins are shown in Table S1. Transfections of siRNA-depleted and control cells were carried out in duplicates with pcDNA3.1-GFP as a transfection or loading control. The human embryonic kidney (HEK) 293 cell line was grown in DMEM in 6-well plates, as described (36). The indicated siRNAs (final concentrations 50 or 100 nM) were combined with jetPRIME (Polyplus) according to manufacturer's recommendations. The mixtures were incubated at room temperature for 20 min before adding to the cells. The cell cultures were split into 12-well plates 48 h later when they received the second siRNA hit together with the splicing reporter construct. The cells were harvested 24 hr later for RNA and protein extraction. Total RNA was isolated with TRI-reagent (Ambion), treated with DNase I (Promega) and transcribed using the Moloney murine leukaemia virus reverse transcriptase (RT; Promega) and primer d(T)_20_ according to the manufacturers’ recommendations. RT-PCR reactions were performed using minigene- and vector-specific primer combinations (Table S1) (14). RT-PCR products were separated by gel electrophoresis and their signal intensities were measured as described (37) to obtain mean exon inclusion levels and their variability.

### Immunoblotting

The cells were washed with PBS and lysed in the RIPA buffer (150 mM NaCl, 1% NP-40, 0.5% deoxycholate, 0.1% SDS, 50 mM Tris, pH 8.0). Protein lysates were loaded onto 10% SDS-PAGE, transferred to nitrocellulose membranes and incubated with antibodies against SRSF1 (Abcam, ab38017), SRSF7 (Protein Tech Group, 11044-1-AP), Tra2β (Abcam, ab31353), c-*myc* (Sigma, PLA0001), GFP (Abcam, ab290), GAPDH (Novus, mb300-322). Secondary antibodies were purchased from Thermo Fisher (#31458). Proteins were detected using the Pierce ECL Western Blotting Substrate (Thermo Fisher) according to the manufacturer's instructions.

### Analysis of enhanced UV crosslinking and immunoprecipitation data (eCLIP)

Published eCLIP data were obtained from ENCODE as GRCh38 (hg38) narrowPeak files (38). eCLIP samples and proteins are detailed in Dataset S2. Samples that passed ENCODE quality standards (*n* = 450) were selected for enrichment analysis, representing eCLIP signals for 150 proteins and 225 cell line/protein pairs, all in duplicate. For each eCLIP accession, exonic sequences corresponding to canonical EF-hand binding sites were assessed for whether they were enriched for eCLIP high-confidence peaks. The enrichment of peaks within EF-hand binding sites was assessed with GAT, v. 1.3.6 (39), using 200 000 simulated samples, randomizing eCLIP peaks within the transcribed genome. Multiple test correction was applied following the Benjamini-Hochberg procedure and samples with corrected *q*-values <0.01 were considered significantly enriched.

### RNA secondary structure predictions

For multiple sequence alignments of canonical EF-hands, we employed the updated RNAalifold with RIBOSUM scoring (v.2.4.18) (40) using minimum free energy and partition functions while avoiding isolated base pairs. Cumulative base-pairing probabilities (BPP) and entropy (S) values were computed for each position of 36-nt long segments encoding canonical EF-hand loops and flanking sequences.

### Bioinformatics and statistics

Overlap between EF-hand loop exons and high-confidence *N*^6^-methyladenosines (m^6^A) was tested using GAT (39). Collections of tentative human m^6^A sites were obtained from most comprehensive reports to date, totaling to 178 049 (41) or 36 556 (42) methylated adenosines.

Relative synonymous codon usage (RSCU) and codon adaptation index (CAI) (43) were computed using the CAIcal utility (44). RSCU value for a codon is the observed frequency of that codon divided by the frequency expected under the assumption of equal usage of synonymous codons (43). The expected CAI values for the query sequences were calculated by generating random sequences with similar GC content and amino acid composition. The expected CAI provides threshold values for discerning true codon preferences from artifacts arising from GC and/or amino acid composition of query sequences.

Branch sites of CBS-encoding exons were predicted with the SVM-BP algorithm (45) using intronic sequences upstream of their 3′ss. Sequence logos were created with WebLogo (46) using alignments generated by Clustal algorithms (47) and checked manually. Fractions of negatively charged amino acids in SMART protein domains (48) were obtained from RACCOON (49).

Inclusion levels of exon 4b following transfection experiments was tested by one-way analysis of variance (ANOVA), followed by Dunnett's post-hoc tests. To compare the ESE/ESSseq scores in the indicated groups and ESEf/ESSf or ESEc/ESSc ratios for codons for Ca^2+^-coordinating residues with the remaining residues in the same exon, we used one-way ANOVA, unpaired two-tailed *t*-test or the Wilcoxon-Mann-Whitney test.

## RESULTS

### ESE/ESS profiles across exons that encode calcium binding sites

**Figure 1.**
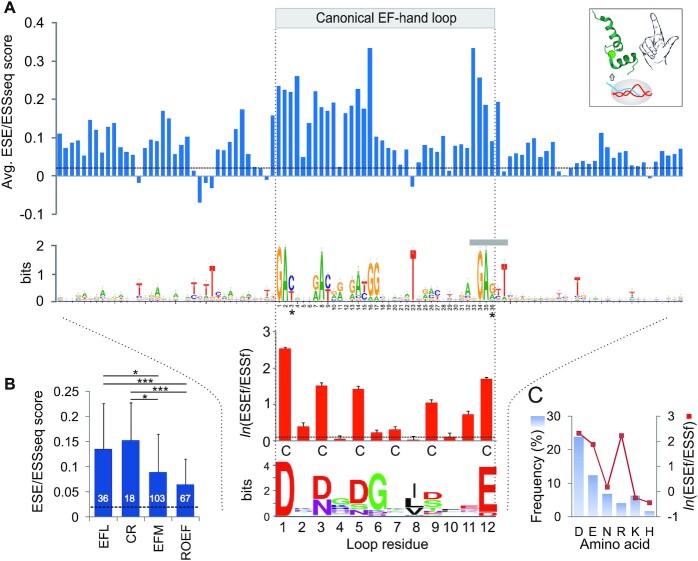
ESE/ESS profiles across human canonical EF-hands. (**A**) ESE/ESSseq scores (*top*) and *ln*(ESEf/ESSf) ratios (*bottom*) across exonic sequences for EF-hand motifs. Higher values predict higher exon inclusion in mature transcripts (6,14). Columns denote means for 296 EF-hands (Dataset S1). Horizontal dashed lines indicate mean ESE/ESSseq scores and *ln*(ESEf/ESSf) ratios for control human exons devoid of splice-site consensus motifs. Logo character height specifies the information content for nts (maximum is log_2_4 = 2 bits) or amino acids (log_2_20 = 4.3 bits). Error bars for *ln*(ESEf/ESSf) values are standard errors of the mean. Asterisks at wobble positions of codon 1 and 12 denote a codon usage bias (Table 1 and Dataset S3) as compared to standard human codon tables (163,164). A grey bar denotes a complete lack of ESS hexamers starting at nt position 33, *ie*. the greatest excess of ESEs over ESSs in the EF-hand motif. Inset (*top right*) exemplifies a canonical EF-hand in Ca^2+^-binding protein 7 (CaBP7; PDB access code 2LV7); sphere denotes Ca^2+^. (**B**) Comparison of average ESE/ESSseq scores for codons encoding EF-hand loops (EFL), their Ca^2+^-coordinating residues (CR), the whole EF-hand motifs (EFM), and EFM devoid of EF-hand loops (‘the rest of EF-hand motifs’, ROEF). Number of nt positions per each group entry is in white. Horizontal dashed line indicates the mean score value for control exons. Asterisks denote significant differences of the means between the groups (*P< 0.01, ***P< 0.0001). (**C**) Frequencies of charged residues and Asn in canonical EF-hand loops (*blue*) and their average exon inclusion potential estimated by *ln*(ESEf/ESSf) values (*red*).

Ca^2+^ binds to over 10 consensus protein motifs, but most frequently to a DxDxDG binding loop (50,51). The best studied and largest DxDxDG group is represented by EF-hand proteins (28,52). The EF-hand motif, first described ∼50 years ago (53), consists of two α-helices bridged by a Ca^2+^-chelation loop (Figure 1A, inset). The loop is traditionally defined by 12 consecutive amino acids, beginning with the first Ca^2+^-coordinating residue, usually Asp, and ending with the last, usually Glu (28,52) (Figure 1A). Ca^2+^ is mainly coordinated by carboxyl oxygens of residues 1, 3, 5 and 12, a backbone amide carbonyl oxygen of residue 7, and an oxygen derived from water bonded to a residue 9 side chain, in a pentagonal bipyramidal geometry (52). The N-terminal part of the EF-hand loop is typically formed by regularly spaced Asp residues, matching the widespread Ca^2+^-binding motif DxDxDG (34).

We first determined ESE/ESS profiles of a nonredundant set of human nt sequences encoding canonical EF-hand motifs (Figure 1A, *top*). These motifs showed a significant increase in predicted exon inclusion levels expressed as mean ESE/ESSseq scores (6) compared with average human exons (Figure 1B). This increase was largely due to a high exon inclusion conferred by codons for Ca^2+^-coordinating residues in the N-terminal EF-hand loop and at codon 12 (Figure 1A,B). In contrast, the average ESE/ESSseq score for hexamers in the rest of the EF-hand motifs was similar to control exons (Figure 1B).

Next, we examined codon-specific splicing activities, expressed as frequency (ESEf/ESSf) or count (ESEc/ESSc) ratios (see the Methods section). The ESEf/ESSf ratios were, on average, higher in the EF-hand loops than in the remaining parts of EF-hand motifs and were most increased for codons encoding Ca^2+^-coordinating residues, with a positional hierarchy of 1>12>3∼5>9 (Figure 1A, *lower panel*). This order reflected their evolutionary conservation, except for conserved Gly at position 6 and Ile/Leu/Val at position 8 (Figure 1A). A similar predominance of Ca^2+^-coordinating codons was found for ESEc/ESSc ratios (Supplementary Figure S1A). When combining all loop codons for Ca^2+^-coordinating Asp, Glu and Asn residues and comparing them with non-coordinating loop counterparts, the mean ESEf/ESSf ratio of the former was ∼5.5-fold higher (*P* < 10^−16^, unpaired two-tailed *t*-test).

We conclude that codons for canonical EF-hand motifs confer higher than average exon inclusion and that this exon-level promotion of EF-hand expression is centred on Ca^2+^ binding. The increase was mainly due to codons for negatively charged residues that dominate the binding loop (Figure 1A-C).

### Splicing activities associated with exonic sequences for noncanonical EF-hands

Noncanonical EF-hand motifs may lack the coordination mechanism outlined above and the composition and length of their binding loops may vary (52). The most diverged subgroup is represented by S100 proteins, which have a unique 14-residue pseudoEF-loop, are exclusively present in vertebrates and often bind additional divalent metals at sites remote to Ca^2+^ (54). These metals include Zn^2+^ and Cu^2+^, i.e. tight I-W series binders that require splice-repressing codons for His and Cys (14), which coordinate both ions (55). Zn^2+^ binding to S100 EF-hands can alter geometry of the Ca^2+^ binding loop and Ca^2+^ affinity (56). S100 interactions with targets differ from canonical EF-hand proteins and, unlike typical EF-hands widely distributed across chromosomes, the majority of human S100 genes map to chromosome band 1q21 (27), in line with their distinct evolutionary history. We therefore examined ESE/ESS profiles of S100 pseudoEF-hands separately from the remaining noncanonical EF-hands.

**Figure 2.**
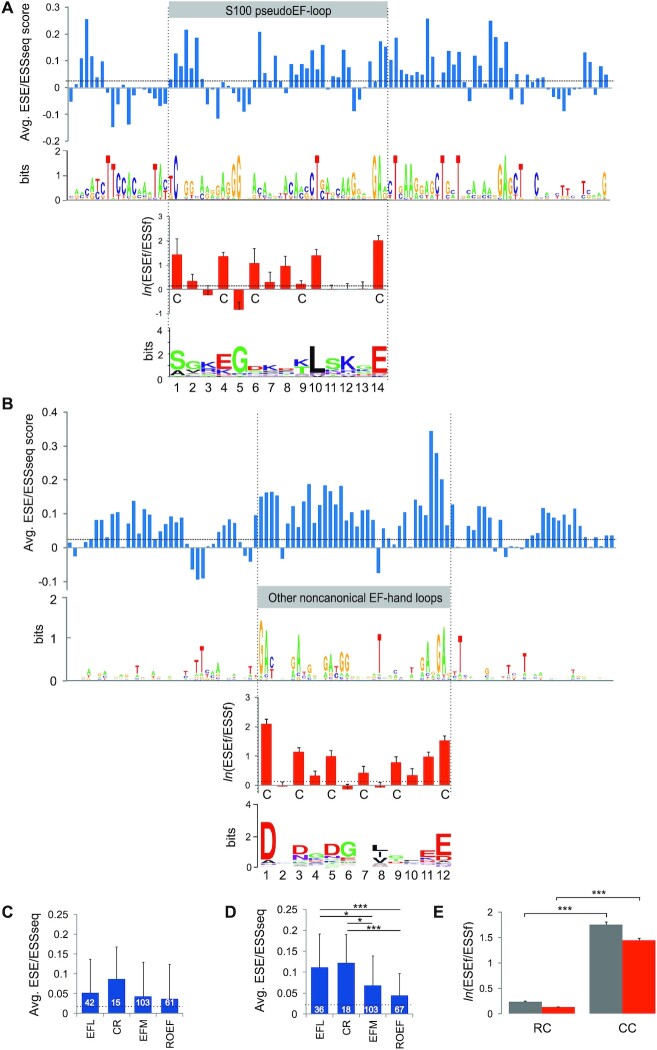
ESE/ESS profiles across human noncanonical EF-hands. (**A**) S100 proteins; (**B**) other noncanonical EF-hand proteins (Dataset S1). For legend, see Figure 1. (C, D) Comparison of mean ESE/ESSseq score values for 15 S100 (**C**) and 66 other noncanonical EF-hand (**D**) loops (EFL), their Ca^2+^-coordinating residues (CR), full EF-hand motifs (EFM), and EFMs devoid of EF-hand loops (ROEF). Asterisks denote significant differences between group means (*P< 0.01, ***P< 0.0001). (**E**) Comparison of mean *ln*(ESEf/ESSf) score values for codons encoding experimentally supported coordinating residues (CC) or the remaining codons in the same exon (RC) in nonEF-hand CBPs (Dataset S1). Grey columns denote solitary Ca^2+^-coordinating codons, red colums are strings of up to five contiguous codons, at least one binding Ca^2+^. Error bars denote the standard errors of the means.

Figure 2A shows that the increase in exon inclusion activities seen for canonical EF-hand loops was not matched by the S100 pseudoEF-loops. Unlike canonical EF-hands, the S100 ESE/ESSseq scores did not significantly exceed the mean ESE/ESSseq score values for average human exons or the values computed for EF-hand sequences outside the pseudoloops (Figure 2C). Even with the limited sample, however, codons for Ca^2+^-coordinating residues in S100 pseudoloops did show slightly higher average ESEf/ESSf profiles than codons encoding the rest of the S100 EF-hand motif (*P* = 0.05). In addition, we observed a marked increase of ESEc/ESSc ratios for the first loop codon (Supplementary Figure S1B). Unlike canonical EF-hands, the first loop position is usually occupied by Ser, which was encoded by UCG codon in over a third of S100 pseudoEF-hands. This codon is the strongest splicing activator (Figure 10 in ref. 14).

In contrast to S100 proteins, hexamer ESE/ESS profiles of the remaining noncanonical EF-hand loops were similar to their canonical counterparts, with an increase over the N-terminal part of the loop and the last loop residue (Figure 2B, D). As in canonical EF-hands, codon-specific scores were centred on Ca^2+^-coordinating residues, showing the same hierarchy of ESEf/ESSf ratios (Figure 2B, D and Supplementary Figure S1C).

Finally, we compared the ESE/ESS profiles for codons for Ca^2+^-binding and non-binding residues in exons that encode CBSs in nonEF-hand proteins. As with EF-hands, predicted splicing activities were significantly higher for codons for Ca^2+^-coordinating residues than for the remaining codons in the same exon (Figure 2E).

Taken together, exonic sequences for canonical and noncanonical EF-hand motifs carry a higher than average probability to be included in mRNAs upon splicing. This property is driven by Ca^2+^-binding residues, which was also observed for nonEF-hand proteins. The exon inclusion levels conferred by noncanonical EF-loop codons were, on average, lower than by canonical counterparts, yet they showed the same hierarchy at Ca^2+^-coordinating codons. In contrast, S100 proteins have a distinct ESE/ESS profile although their close to normal exon inclusion capacity of their pseudoloop sequences might be compensated by the first codon. Thus, the exonic splicing code evolved to safeguard codons for CBSs in mature transcriptomes.

### Codon usage bias at canonical EF-hand loops

Codon usage in eukaryotes is influenced by adaptive forces at the level of pre-mRNA splicing (57). To examine EF-hand loops for such selection pressures, we first computed the codon adaptation index (CAI), a gauge of directional codon usage bias (43). CAI measures similarities between the synonymous codon usage of a gene and synonymous codon frequencies of a reference set. Using both Markov and Poisson methods (44), CAI of the loop sequences was significantly higher than the expected CAI computed for 500 random sequences of the same GC and amino-acid content (avg. 0.86 versus 0.80, *P*< 0.05), providing evidence for selection. At position 1 of the loop, the usage of Asp codon GAC was higher than expected (Table 1). This codon confers, on average, a higher exon inclusion than its underrepresented synonymous counterpart GAU (14). At position 12, however, the frequency of Glu codons was biased towards GAG rather than GAA, which gives somewhat higher exon inclusion (14). GAC and GAG triplets were among codons with the highest average RSCU values alongside with additional two codons common in EF-hand loops (GGC and CUG) while low-RSCU codons included high-exon inclusion NCG triplets (Supplementary Figure S2A).

**Table 1.** Codon usage at main Ca^2+^ coordinating positions of canonical EF-hand loops and their predicted exon inclusion potential

Codon	Amino acid	Codon usage per 10^3^ (counts)	Loop codon 1*	Loop codon 3	Loop codon 5	Loop codon 12*	Average *ln*(ESEf/ESSf)	Fraction in 93 487 human cDNAs (*n*)^a^
GAU	D	107.1 (378)	0.37 (110)	0.26	0.39	0.02	1.51	0.46 (885 429)
GAC	D	130.4 (460)	0.63 (184)	0.32	0.27	0.06	3.15	0.54 (1020 595)
GAA	E	42.8 (151)	0	0	0	0.30 (89)	2.60	0.42 (1177 632)
GAG	E	77.7 (274)	0	0	0	0.62 (182)	1.14	0.58 (1609 975)
AAU	N	34.6 (122)	0	0.18	0.10	0	−0.81	0.47 (689 701)
AAC	N	34.9 (123)	0	0.21	0.05	0	1.16	0.53 (776 603)

^a^Data from human codon usage tables for the standard genetic code (163). Asterisks show significant (*P*< 0.01) deviation as compared to expected proportions. EF-hand loop codon counts and RSCU values are shown in full in Dataset S3.

To examine other possible selection pressures involved in the EF-hand loop codon usage bias, we compared codon usage at EF-hand loop positions 1 and 12 with previously derived codon usage data for genes involved in pattern specification *vs*. mitotic activity (58,59). We found that the loop codon usage bias resembled more codon usage signatures of genes involved in cell differentiation than those involved in proliferation (Supplementary Figure S2B). With a total of 59 synonymous codons of standard genetic code, we observed a positive correlation between the ESEf/ESEf ratio and the fold excess in codon usage frequencies in genes involved in differentiation over proliferation (Supplementary Figure S2C; *r* = 0.62, *P*< 10^−6^). The correlation remained significant with 27 synonymous codons that were enriched in the ‘differentiation’ gene set (green and yellow ovals, Supplementary Figure S2C; *r* = 0.73, *P* = 0.0002), but disappeared for frequencies of 32 synonymous codons enriched in the proliferation gene category (grey oval, *r* = 0.16, *P* = 0.2). Synonymous codons showing the highest overrepresentation in the differentiation *vs*. proliferation sets (58) invariably showed a high exon inclusion capacity (yellow oval, Supplementary Figure S2C).

We conclude that the usage bias toward splice-enhancing codons in the EF-hand loop is selection-driven and is dominated by the first Ca^2+^-coordinating position (Table 1). Codons preferred at loop positions 1 and 12 are also preferred in genes involved in differentiation as opposed to genes involved in mitotic activity (Supplementary Figure S2B, C). The correlation between predicted exon inclusion of synonymous codons and their usage bias toward genes involved in pattern specification suggests that cell differentiation has been an important driving force of ESE/ESS evolution (Supplementary Figure S2C).

### Exon-intron structure of EF-hand motifs and other CBPs

During evolution, many CBPs have evolved domain-specific AS patterns. For example, AS of EF-hands in α-actinins is limited to chordates whereas AS of their actin-binding domains was found in arthropods, nematodes and platyhelminths (60). AS often evolved upon exon duplication followed by exon subfunctionalization, a mechanism particularly common for highly conserved muscle- or energy-related functions, as illustrated for actinins, P/Q-type Ca^2+^ channels or the Ca^2+^-sensitive E1 subunit of the OGDH complex (16,61–63). In these genes, duplicated exons are often separated by short introns that harbour distant lariat-intron branch points positioned away from their usual location near 3′ss, enforcing their mutually exclusive usage (14,64). Regulation of these and other alternatively spliced exons would be expected to rely more on ESEs/ESSs and their respective trans-acting factors as compared to constitutively spliced exons (*eg*.^4^,^65^,^66^).

**Figure 3.**
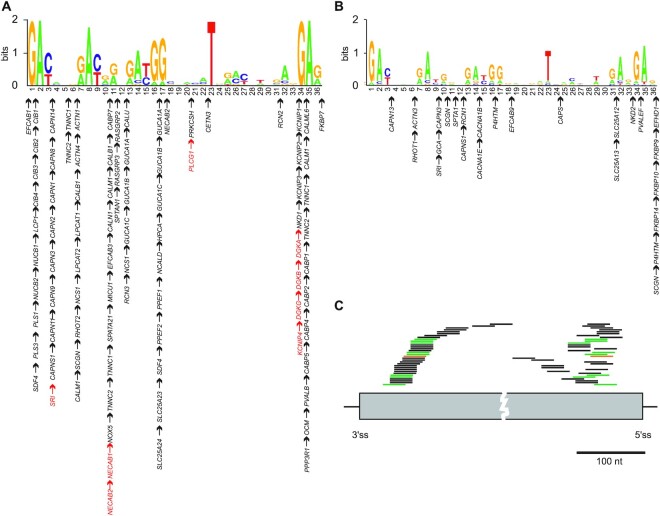
Exon-intron organization of EF-hand loops. (A, B) Exon splits for canonical (**A**) and noncanonical (**B**) EF-hands. Arrows denote exon splits at nt positions of EF-hand loops encoded by the indicated genes. Black arrows denote two-exon splits; red arrows denote canonical EF-hands encoded by three exons, with the loop split between the second and third exon. Their observed and expected numbers are in Table 3. (**C**) Locations of one-exon EF-hand loops relative to splice sites. Grey box denotes an exon, flanking horizontal lines are introns, and vertical lines are splice sites. Each loop is represented by two bars: bars on the *left* show distances to exon 3′ss (sorted); bars on the *right* show their distances to 5′ss of the same exon. Nucleotide logos for one-exon and ≥2-exon EF-hand loops are in Supplementary Figure S3. Bars for canonical EF-hand loops are in black (*n* = 24), non-canonical loops in green (*n* = 8) and a S100 loop (n = 1) in orange. EF-hand loops are shown only for exons smaller than 400 nt.

Consistent with this expectation, intronic sequences adjacent to exons that code for canonical, noncanonical and nonEF-hands were similar to controls (Supplementary Figure S3A, B). Their traditional splicing motifs did not appear to have been influenced by intronic insertions that split the Ca^2+^-binding loop (Supplementary Figure S3A, B). The strength of their yUnAy consensus (67) branch sites and their distances from 3′ss were also similar in these groups (Supplementary Figure S3C,D) as was the intrinsic strength of their splice sites (Supplementary Figure S3E). A variant GC 5′ss, which are present in ∼1% of human introns (68) and require more help from ESEs than GT 5′ss (69), was found in EF-hand motifs encoded by single exons in *EFCAB9*, *EFHB* and *NOX5* (Supplementary Figure S3A). Most canonical or noncanonical EF-hand motifs were, however, encoded by at least two exons (Table 2). These exon splits were found in the Ca^2+^-binding loop more often than expected (42.7% versus 33.3%, Table 3). Moreover, the splits were not random (*P*< 10^−16^, χ^2^ test): they accumulated in the N-terminal part of the chelation loop at 10 different positions and around codon 12 at 4 different positions, with only very few intronic insertions between the two clusters (Figure 3A). Some positions were dominated by specific EF-hand subfamilies: for example, calpain genes (*CAPNx)* showed EF-hand exon splits just after the first codon of the loop while genes for guanylate cyclase-activating proteins *(GUCAx)* were split within the first and second position of the conserved Gly codon 6 (Figure 3A). Exon splits detected in the C-terminus of the loop are exemplified by cytosolic voltage-gated potassium channel-interacting proteins (*KCNIPx* genes) as well as *DGKx* genes, which code for the diacylglycerol kinase family. The predominance of intronic insertions in the N-terminal half of the binding loop and around the last position was also seen for noncanonical EF-hands (Figure 3B). Exons split within the loop had similar nucleotide frequencies at each loop position as those outside the loop (Supplementary Figure S4). The ESE/ESSseq scores for EF-hands encoded by 2 or more exons tended to be somewhat lower compared with one-exon EF-hands (Supplementary Figure S5). Finally, distribution of unsplit EF-hand loops within a single exon is shown in Figure 3C.

**Table 2.** Exon-intron structure of canonical and noncanonical EF-hand motifs

Number of exons^a^	1	2	3 or more
Number of canonical EF-hands	79	178	39
Number of noncanonical EF-hands	15	47	4
Total	94	225	43

^a^χ^2^ for 2×3 contingency table: 3.7 (*P* = 0.16). S100 pseudoEF-hands split into 3 or more exons were not found in our sample.

**Table 3.** Number of EF-hand motif exon splits

Observed/expected for random sequence	Within the loop	Outside the loop
Canonical (n = 213)	87/71	126/142
Noncanonical (n = 49)	25/16.33	24/32.67
Both (n = 262)^a^	112/87.33	150/174.67

^a^ *P*<0.001, binomial test, assuming fixed lengths of the loop (36 nt) and the full EF-hand motif (108 nt).

Taken together, in contrast to ESEs/ESSs at CBSs (Figures 1 and 2), intronic sequences flanking exons encoding CBSs are similar to controls (Supplementary Figure S3). Intronic insertions separating EF-hand loops into two or three exons clusterred in the N-terminus and around the last Ca^2+^-coordinating residue (Figure 3A,B).

### mRNA secondary structure and canonical EF-hand motifs

**Figure 4.**
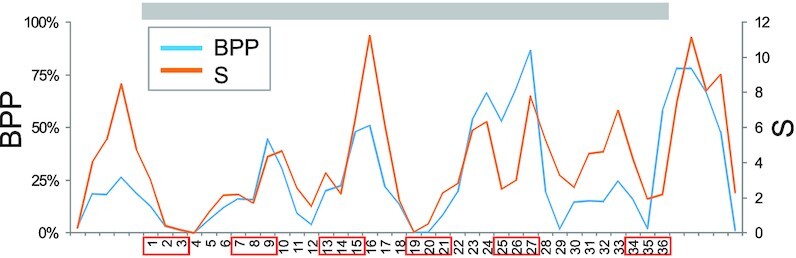
mRNA base-pairing probabilities across the EF-hand loop of Ca^2+^-binding CABPx proteins. BPP, cumulative base-pairing probabilities, S, entropy. Grey rectangle denotes the EF-hand loop, red boxes denote codons for Ca^2+^-coordinating residues. Nt sequences encoding the 12-amino acid EF-loop consensus are numbered from the first position of the first codon. BPP/S values for additional CBPs are in Supplementary Figure S6.

Exonic segments coding for EF-hand loops are purine-rich (Supplementary Table S3). Alignment of 36-nt sequences encoding 296 canonical or 66 noncanonical EF-hand loops (excluding S100 proteins) revealed a set of alternating GA dinucleotides at codons 1, 3, 5, 9, 11 and 12, i.e. largely Ca^2+^-coordinating residues (Figure 1). As compared to ESSs, the GA dinucleotide is strongly overrepresented in ESEs where it shows the second highest enrichment after the CG dinucleotide (Supplementary Figure S6A, B). GA dinucleotides encoding residues in the N-terminal part of the chelation loop are regularly spaced, which might shape mRNA secondary structure and contribute to a fold in common. To test this, we employed RNAalifold (40), a comparative method that predicts a shared structure for a fixed input alignment of RNA sequences. RNAalifold minimizes the overall free energy while using information on compensatory mutations between a set of aligned exons. Although the nucleotide diversity of even canonical EF-hands was too high to reveal a structure in common *ab initio*, some subfamilies, including *CABPx* genes and those encoding CTER or CPV core proteins (28), showed a consistent decline in base-pairing probabilities toward the wobble position of the first Asp codon (Figure 4 and S7). This position is thus more likely to be unpaired and more accessible for interactions with ligands. The decline was not consistent for other Ca^2+^-coordinating codons, suggesting that the accessibility of the first codon could be particularly important for exon recognition by the spliceosome. The first Asp residue plays a critical role in Ca^2+^ binding (35), shows the highest conservation in the EF-hand loop (Figure 1A, 2B) and when mutated in the reporter construct, it also showed the greatest exon repression along with Asp codon at position 5 (*see below*). Together, these predictions support an increased accessibility of the wobble position of the first Asp codon to ligand binding, in line with single-stranded contexts having a stronger effect on splicing than those located in double-stranded regions (70).

### Regulation of a DxDxD exon by SR proteins

So far, we have shown that EF-hand motifs have a higher than average capacity to include their exon sequences in mature transcripts and that this property is mediated by Ca^2+^-coordinating codons in the loop, which accumulated exon splits in two clusters (Figure 1-3). In our sample of canonical EF-hand loops, almost a quarter of all residues were Asp, with Glu contributing over one-eighth to the total while charged amino acids accounted for ∼47% residues (Figure 1C). Asp (GAY, where Y is pyrimidine) and Glu (GAR, where R is purine) codons share GA dinucleotides, which are overrepresented in ESEs as compared to ESSs, particularly when followed by cytosine or adenosine (Supplementary Figure S6A-C). What ligands do they bind to?

**Figure 5.**
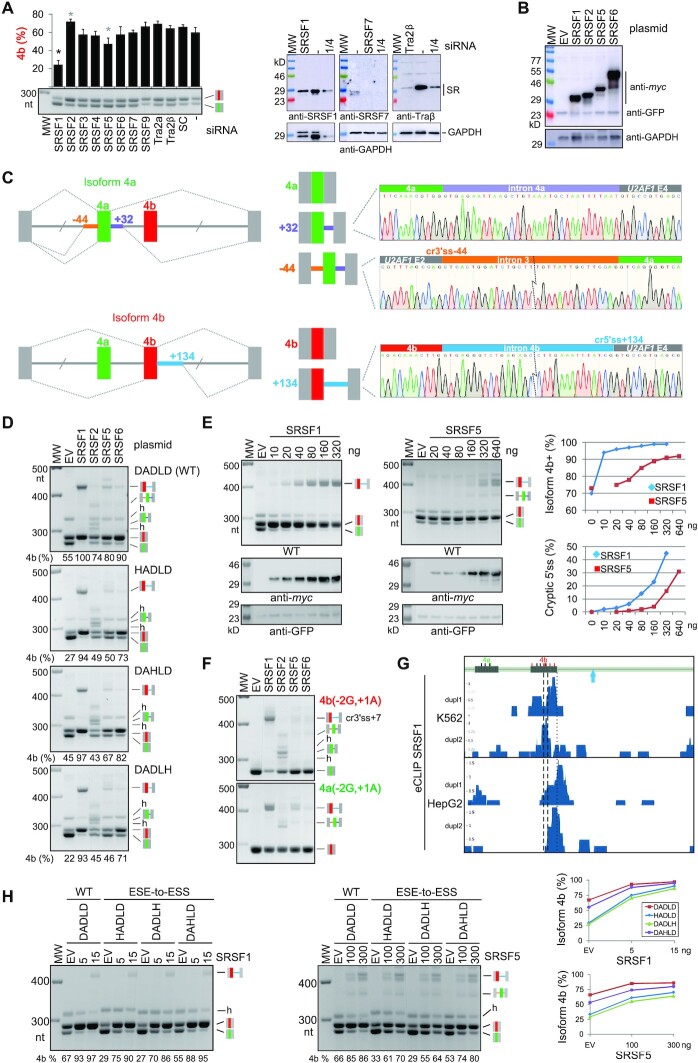
Identification of SR proteins involved in regulation of a DADLD site in OGDH. (**A**) Antagonistic effects of SR proteins on *OGDH* exon 4b inclusion. Significant differences (*P*<0.05) are shown by black (one-way ANOVA followed by Dunnett's post-hoc test) or grey (pair-wise comparisons) asterisks. Columns denote means, error bars are SDs; SC, mixture of scrambled controls, MW; molecular weight marker. Representative immunoblots are shown to the *right*. Antibodies are shown at the *bottom*. **(B−D)** Overexpression of a subset of SR proteins induces cryptic 5′ss. (**B**) Immunoblot with the indicated SR expression constructs (*top*) and antibodies (*right*). (**C**) Schematics of canonical (*above each pre-mRNA*) and aberrant (*below*) splicing. Sequence chromatograms of aberrant transcripts are to the right. (**D**) Transient cotransfections of WT and mutated *OGDH* reporters (*right*) with plasmids expressing the indicated proteins (*top*). Relative expression of canonical isoform 4b is shown at the *bottom*; mRNA products are shown to the *right*; h, heteroduplex. DADLD substitutions are shown in Supplementary Table S1. (**E**) Comparison of SRSF1 and SRSF5 to promote isoform 4b and activate cryptic 5′ss. Empty vector (EV) was added to 320 ng (SRSF1) or 640 ng (SRSF5). Signal measurements are shown to the *right*. (**F**) Inactivation of exon 4b (*top panel*) or exon 4a (*bottom panel*) by mutation. Substitutions (relative to splice sites) are shown in *red* or *green*, respectively. (**G**) eCLIP signals for SRSF1 in *OGDH* in two cell lines (38). *Blue* arrow indicates cryptic 5′ss induced by SRSF1/SRSF5. *Dashed* vertical lines flank the DADLD motif and a *dotted* line marks authentic 5′ss of exon 4b. Short vertical bars at the top denote GA dinucleotides in each exon, their *red* and *green* versions denote GA dinucleotides lost and gained following the exon duplication event, respectively, assuming that exon 4a originated from exon 4b (14,16), (**H**) ESE-to-ESS conversions at each Asp of the DADLD motif do not significantly alter the SRSF1- or SRSF5-induced activation of the Ca^2+^-sensitive isoform. Amounts of expression plasmids (ng) are at the *top*. Mean exon 4b inclusion levels shown at the *bottom* of each gel are plotted to the *right*.

Supplementary Table S2 shows a compilation of RBPs that bind motifs containing GAN codons. They include SR proteins that preferentially contact GA-containing targets, such as SRSF1, SRSF2, SRSF4 and SRSF5 (71–75). To test their role in the maintenance of exons coding for common Ca^2+^-binding sites, we first employed the *OGDH* splicing reporter with mutually exclusive exons 4a and 4b to examine their usage in HEK293 cells individually lacking most SR proteins (Figure 5A). *OGDH* exon 4b encodes a core DxDxD motif, which is essential for OGDH activation by Ca^2+^ and bears similarities to EF-hand motifs (16,32). The screening revealed the highest exon 4b skipping in cells depleted of SRSF1 (also known as ASF/SF2; Figure 5A). We also observed minor exon 4b skipping in cells depleted of SRSF5 (SRp40) and the opposite response upon SRSF2 (SC35) depletion. In contrast to knockdown, overexpression of SRSF1 or SRSF5 increased exon 4b inclusion (Figure 5B−E). However, overexpression of both SRSF1 and SRSF5 also activated intron-proximal cryptic 5′ss, namely in intron 4a (SRSF5) and intron 4b (both SRSF1 and SRSF5). The SRSF1- or SRSF5-induced increase in the relative expression of the Ca^2+^-sensitive isoform 4b was thus kept in check by activating cryptic 5′ss that lead to unproductive transcripts (Figure 5C, D). The enhancement of SRSF1- and SRSF5-mediated exon 4b inclusion appeared to be similar, despite ∼4-fold higher expression of SRSF1 (Figure 5E).

To establish if the two SR proteins act on exon 4b or 4a, we inactivated each mutually exclusive exon by splice-site mutations (Figure 5F). Upon inactivation of exon 4b, SRSF1 overepression induced a proximal 3′ss 7 nts downstream of authentic 3′ss besides cryptic 5′ss seen in the WT, maintaining most of exon 4b in the mRNA, including the DADLD site (Figure 5F, *top*). Following inactivation of exon 4a, SRSF1-induced cryptic splice-site was not affected (Figure 5F, *bottom*, and Figure 5D). Similarly, SRSF2-induced cryptic sites were used upon inactivation of each exon. Thus, both SR activators facilitated the inclusion of exon 4b and DADLD motif in the absence of authentic splice sites, employing cryptic sites with mostly lower intrinsic strengths (Supplementary Table S4, Figure 5C).

Inspection of public eCLIP data (38) showed accumulation of SRSF1 peaks in *ODGH* exon 4b and their lack in exon 4a (Figure 5G). In most replicas, the peak signals were closer to authentic 5′ss than the DADLD motif (Figure 5G). To test if ESE-to-ESS mutations that inactivate the DADLD site influence SRSF1- and SRSF5-mediated exon 4b inclusion, we individually cotransfected each expression construct with the WT and mutated *OGDH* reporters following titration, but we observed no reduction in the SR-induced switch to isoform 4b by DADLD mutants (Figure 5H).

Taken together, we identified SR proteins that control relative expression of the DADLD motif and Ca^2+^-mediated activation of the OGDH complex. SR proteins with two RRMs (SRSF1 and SRSF5) increased the inclusion of DADLD-encoding exon, acting in the opposite direction as compared to one-RRM SRSF2. The apparent functional redundancy of SRSF1 and SRSF5 at canonical splice sites could be dissected by examining SR-induced cryptic splice sites of the same exons, revealing distinct function of the two proteins in *OGDH* splicing.

### RBPs that preferentially bind exons encoding EF-hand loops

**Figure 6.**
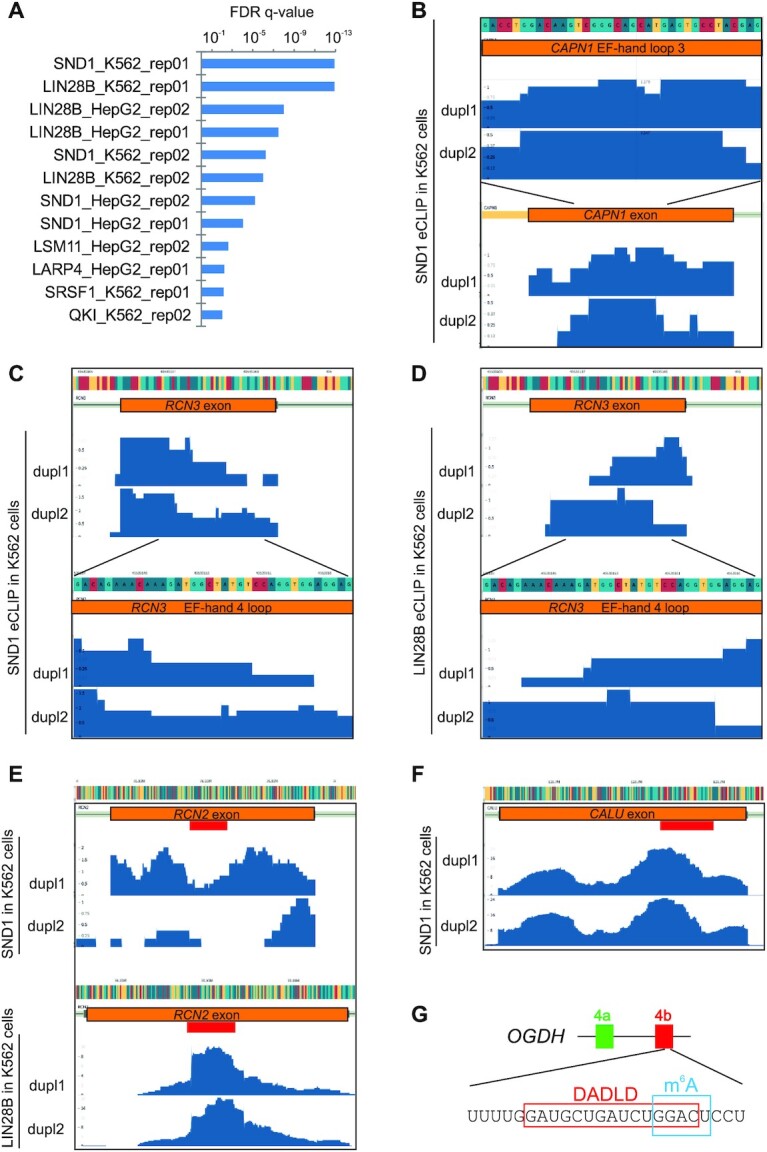
RBPs that bind to exonic sequences encoding EF-hand loops. (**A**) RBPs implicated by GAT (39) as preferred binders to exonic segments coding for canonical EF-hand loops. Full list of tested RBPs and their corrected *q*- and *P*-values is in Dataset S2. (**B**) Example of SND1 eCLIP binding to calpain 1 (*CAPN1*) exon. The exon encodes the Ca^2+^-binding loop of the third EF-hand motif (nt sequence is at the *top*). (C−F) Examples of SND1/LIN28B binding to exons that code for EF-hands in the CREC protein family (106). SND1 (**C**) and LIN28B (**D**) binding to *RCN3* exon that encodes EF-hand 4 of reticulocalbin 3; (**E**) SND1/LIN28B binding to a reticulocalbin 2 exon. *Red* rectangle denotes the canonical EF-hand loop; (**F**) SND1 binding to calumenin (*CALU*) exon that encodes EF-hand motif 2. (**G**) m^6^A-modified (41) GAC codon for the last Asp residue in the DADLD motif. The motif is essential for OGDH activation by Ca^2+^ (32).

To identify RBPs that contact exonic segments encoding EF-hand loops more systematically, we examined the largest eCLIP dataset (38). Using GAT (39), we compared observed and expected colocalization of canonical EF-hands and eCLIP peaks, ranking eCLIP signals of 150 proteins across 79 one-exon and 217 split-exon canonical EF-hands (Datasets S1 and S2). Twelve eCLIP samples showed a significant overlap between exonic segments for canonical EF-hands and those with positive eCLIP signals, including SRSF1, but only two proteins, SND1 (staphylococcal nuclease domain containing 1) and LIN28B, were consistently detected in two replicas and independent cell lines (Figure 6A, Dataset S2). Because conserved GA-rich motifs encode the ends of canonical binding loops (Figure 1A), we varied the size of padding added to the first and last codons in each direction (3, 6 and 9 nts), but the enrichment of SND1 and LIN28B remained robust in each case.

Figure 6B-F shows examples of positive eCLIP signals seen for the CERC family of CBPs (*RCN1, RCN2, RCN3*,and *CALU*). Other positive CBPs included nucleobindins, calpains, calmodulin and microtubule acting crosslinking factor 1 (also known as macrophin 1). A subset of positives exhibited sharp declines at splice sites (Figure 6B-F), in line with the majority of eCLIP peaks for both proteins in the coding sequence (38 and refs. therein). The enrichment for SND1 and LIN28B remained significant with a reduced collection of loops encoded by 2 or more exons, suggesting that the eCLIP signal is conditional on splicing. We also noticed that LIN28B and SND1 displayed a mutually exclusive pattern of binding to some EF-hand loop exons and that there was also a significant exon overlap (*P*< 0.001, χ^2^ test) for the two proteins (Figure 6C,D), suggesting that they may occupy competing complexes. Together, these data suggest that SND1 and LIN28B are preferentially recruited to exonic segments that encode a subset of canonical EF-hand loops, in agreement with binding preferences for GA-rich targets of both SND1 (76) and a LIN28B paralogue, LIN28A (77).

## DISCUSSION

### How pre-mRNA splicing facilitated Ca^2+^ signalling

The metal-binding complement of eukaryotic proteomes comprises at least a third of all gene products (78–80). The number of human proteins predicted to bind Ca^2+^ or Mg^2+^ has been estimated at ∼10^5^ each, contributing over 60% to the total (80), strongly suggesting that binding sites for these alkaline earth metals are encoded by a large proportion of exons. These coding sequences are essential for many cellular functions and have a priviledged position in eukaryotic transcriptomes, particularly those involved in regulation of Ca^2+^ as the most important intracellular second messenger (20). CBPs, including EF-hand proteins, were found in prokaryotes (81,82), but expanded a great deal in unicellular eukaryotes, which evolved not only the first intracellular Ca^2+^ stores and release channels (83) but also introns, some of them extraordinarily short (84). In higher eukaryotes, the number of Ca^2+^-binding domains including widespread EF-hand core motifs massively increased (18,34), but molecular mechanisms underlying their expansion and evolutionary success have remained poorly understood.

Our results demonstrate the importance of ESEs for the mRNA inclusion of CBSs. The function of ESEs to maintain coding segments in mature transcriptomes was underappreciated in the past, with intron removal mainly viewed as splice-site and branch-site driven. Although this holds true for short introns in lower organisms such as yeasts, primary transcripts of higher eukaryotes are dominated by long introns (85). Their exons rely much more on cross-exon interactions between degenerate 3′ss and 5′ss during early spliceosome assembly, known as exon definition (86,87). For example, both human and *Arabidopsis* splice sites provide only ∼50% or less of the information necessary for accurate intron excision, with branch sites contributing only marginally (85). This is consistent with the finding that traditional splicing signals in introns that flank exons encoding CBSs are broadly similar to the average (Supplementary Figure S3). We could not completely exclude a possible deviation in the polypyrimidine tract of noncanonical EF-hand exons (Supplementary Figure S3A,B), which may affect pre-mRNA contacts with U2AF65 or other uridine- or cytosine-binding RBPs (36,88). In *Xenopus*, uridines located at a similar distance from 3′ss were not efficiently bound to U2AF when converted into pseudouridines (89), suggesting that the RNA modification at these positions is important for U2AF65 interactions.

**Figure 7.**
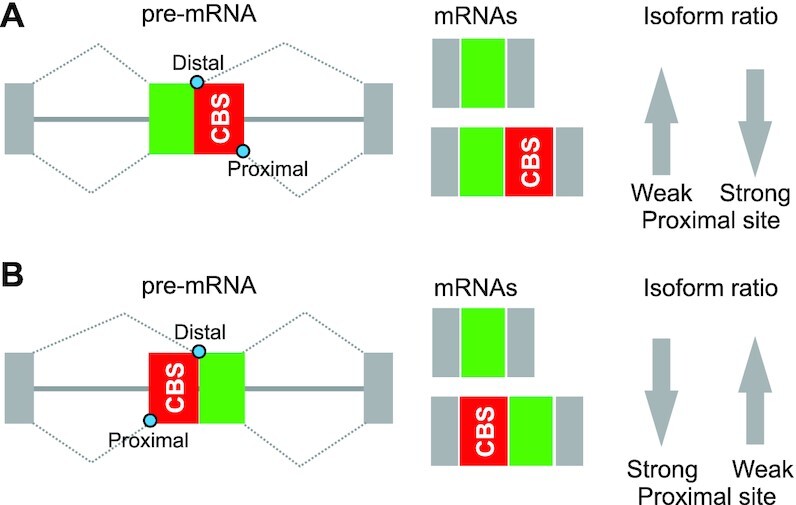
Promotion of exon inclusion by codons for CBSs. Exons are shown as boxes, introns as lines. CBS, Ca^2+^-binding site encoded by an alternatively spliced exonic segment. *Blue* circles denote alternative 5′ss (**A**) and 3′ss (**B**). Arrows indicate expected relative changes in the balance of CBS-including and -excluding mRNA isoforms, reflecting the strength of competing splice sites. The scheme would be analogous for other types of AS, including mutually exclusive exons (Figure 5H) or cassette exon inclusion.

In contrast, auxiliary splicing motifs in exonic segments for CBSs elicit significantly higher exon inclusion levels as compared to average exons (Figures 1 and 2). This increase is largely due to the excess of codons for Ca^2+^-binding residues, both in EF-hands and nonEF-hands, and is mainly afforded by codons for negatively charged amino acids (Figures 1 and 2). The exon-level promotion by codons for these residues is context-dependent, *ie*. not unconditional. For example, the inclusion of identical CBS sequences in mature transcripts would be greater if their proximal splice sites are stronger than their competing distal counterparts (Figure 7). Similarly, the inclusion enhancement would be greater for alternatively spliced exons as compared to constitutive exons, facilitating adoption of new sequences in mRNAs. Hence, the relative contribution of CBSs to variation in mammalian codon usage is also likely to be exon-specific.

Negatively charged residues are strongest in orienting water molecules toward protein surfaces and are thus most beneficial for protein solubility (90), the property absolutely critical for life based on poorly soluble Ca^2+^ phosphates (91). Solubility can be elevated or suppressed by mutation while preserving protein stability and function (92), suggesting that such optimization took place during evolution. Frequencies of negatively charged amino acids in proteins are substantially higher than those expected for the relative number of codons in the standard genetic code and the average number of codons per acidic residues is lower than that per basic amino acids (93,94). In contrast, frequencies of Cys and His codons, which generally repress exon inclusion (14) (Figure 5H), are below expected levels (93,94). This bias was found both in eu- and prokaryotes (94), in line with only a minor overall enhancement of exon inclusion by the underlying codons (Figure 1B, 2D). Negatively charged residues are also required for Ca^2+^-binding of intrinsically disordered proteins where the requirement for regular Asp spacing does not appear as stringent as in EF-hand proteins (95). As in EF-hands, replacement of essential Asp residues by Glu in disordered proteins can reduce Ca^2+^ binding (95). Negatively charged residues are also more common in exposed linkers than in globular domains (49), but are depleted from protein-nucleic acid binding interfaces (96).

It was suggested that the evolutionary history of (Dx)_n_ repeats as core Ca^2+^-binding motifs may be similar to that of (RS)_n_ and methylated (RG)_n_ dipeptides, possibly involving a slippage mechanism for hexamer repeat expansion (34). The (RS)_n_ motifs are common in the RS domains of SR proteins (97) while methylated (RG)_n_ repeats bind the Tudor domain, as exemplified by SND1 binding to spliceosomal Sm proteins (98). Most codons for the two charged amino acids at position 1 of these dipeptides (Asp and Arg) have very high ESEf/ESSf ratios (Figure 1C), including three Arg codons (CGA, CGG, and CGC) (14). This supports a shared role of ESEs in the evolutionary expansion of (Dx)_*n*_, (RG)_*n*_ and (RS)_*n*_ signatures, providing them with a small but detectable protection from being spliced out.

Ca^2+^ binding to EF-hands can lead to a major change in conformation (e.g. in calmodulin), less profound changes (S100 proteins) or induce no or only minimal structural alterations (parvalbumin) (52). For example, in the absence of Ca^2+^ the high-affinity NOX5 EF-hand domain is unfolded and detached from the rest of the protein, but acquires an ordered and compact structure upon Ca^2+^ binding (99). This conformation enables Asp-mediated contacts with the enzyme dehydrogenase domain (99). EF-hands do not only serve as Ca^2+^-binding sites, but also as dimerization motifs and in multidomain proteins EF-hands can mediate interactions with other domains (52). They tend to occur in pairs, forming a four-helix bundle stabilized by a short antiparallel β-sheet between the pairs’ loops and this pairing appears to be pervasive and conserved (52). Thus, the capacity of Asp codons to maintain higher than average inclusion levels of EF-hand coding sequences may not be limited to Ca^2+^ coordination in the loop, but could extend to other regulatory contacts, further supporting the evolutionary expansion of EF-hands. Overall, our results indicate that expression of acidic amino acids is promoted at the exon level to safeguard protein binding sites for Ca^2+^. They also show that extensive coupling between separate gene expression steps (100) goes beyond the first round of translation and involves exon-specific metallome constraints hardwired into the auxiliary splicing code.

### Exon-intron structure of EF-hands and ESE/ESS profiles across the Ca^2+^-binding loop

Cellular compartments in which a protein folds can override its binding preferences and keep competitive metals out of incorrect proteins (79). Eukaryotic cell compartments often rely on distinct function of alternatively spliced mRNA isoforms. AS involving EF-hands, their linkers or outlying residues may produce protein isoforms with distinct Ca^2+^ sensitivities (60,61,101–107). The preferential insertions of introns within the EF-hand loop (Table 3) where the exonic splits cluster in its N-terminal portion and around the last loop position (Figure 3A,B) separate cation-coordinating residues at the exon level (Figure 3A, B). Apart from facilitating their expansion by exon duplication during evolution, such separation would allow the cell to test their new permutations and properties, including Ca^2+^ affinities and Ca^2+^*vs*. Mg^2+^ selectivities (52,108), if alternatively spliced.

The lowest ESE/ESSseq score in both canonical and noncanonical EF-hand loops was observed for highly conserved uridine at codon 8 (Figure 1A and 2B), which separates the two intron insertion clusters (Figure 3A,B). This raises a possibility that the uridine contributed to the ‘split cold spot’ between the N-terminal half and the beginning of the exit helix by repressing the emergence of new splice sites ∼5−7 nts in each direction (Figure 3A, B). Similarly, uridines preferred just upstream and downstream of loop codons 1 and 12, respectively, are likely to dampen the high ESE/ESS profiles at these positions (Figure 1A). For example, the exon inclusion potential predicted by hexamer ESE/ESSseq scores around position 1 is reduced by three adjacent conserved uridines. The UUUGAY hexamers are splicing-neutral (6), with ESE activities gained only by adding nts 3′ of the Asp codon (Figure 1).

### Codon usage bias in EF-hand loops

Synonymous codons do not carry equal probabilities to promote exon inclusion by AS (6,9,109,110). Codon usage is not uniform across exons and codon preferences vary with distance from splice sites (57,111). For example, a preferential usage of GAA versus synonymous GAG codon was observed in the proximity of splice sites (57), which is consistent with somewhat larger mean distances between splice sites and GAG codon 12 than GAA codon 12 in our sample of canonical EF-hand loops. GAA triplets bestow, on average, higher exon inclusion levels than GAG counterparts (Supplementary Figure S6C) (6,14). The selection pressure to preserve ESEs is strong and constrains the evolution of a substantial proportion of coding nts (11). The preferential usage of the splice-enhancing GAC codon over synonymous GAU at key position 1 of the EF-hand loop (Table 1) would provide a mechanistic explanation for this constraint, in agreement with evidence for selection against exon inclusion-reducing mutants, demonstrating that evolutionary pressures at wobble positions can uphold efficient splicing of important exons (8).

Codon usage is also markedly influenced by dichotomous choices for metazoan cells to proliferate or differentiate (58,59). These decisions are, however, controlled by AS (112) and by the auxiliary splicing code in exons (Supplementary Figure S2C). The codon usage bias in our sample of canonical EF-hands was intermediate between signatures typical of proliferation and differentiation genes, but was skewed more toward the latter category (Supplementary Figure S2B). This would be expected given the involvement of many CBPs in both processes and their pivotal role in differentiation, particularly into neural and muscle cell types (*eg*. 113).

Another potential source of codon usage bias is differential codon susceptibility to premature translation termination (114). Notably, codons required to bind common divalent metals in the I-W series encode both ‘fragile’ (Glu and Cys) and ‘robust’ (Asp and His) residues (Supplementary Table S5). Fragile residues are exclusively encoded by fragile codons, which can be changed to a nonsense codon by a single-nt substitution, as opposed to robust codons, which require at least 2 point mutations (114). Avoiding fragile codons and using robust synonymous codons instead might therefore reduce the risk of premature termination at metal binding residues. Indeed, robust codons were preferred at positions 1 and 12 of the canonical EF-hand loops (Supplementary Tables S1 and S5) as well as at codon 6 for conserved Gly (Figure 1 and S8A). The fragile GGA codon for Gly residue was also depleted in genes involved in differentiation as compared to genes involved in mitotic cell division (Supplementary Figure S8A) (58).

Apart from global synonymous codon selection pressures such as GC content, secondary structure, mutation and drift, ESEs/ESSs, translation efficiency, sense-to-nonsense codon susceptibility and cellular differentiation, potential local factors cannot be excluded (115), including those related to Ca^2+^ binding. Ca^2+^ ions are initially bound by EF-hand ligands in the more flexible N-terminal part of the loop (positions 1, 3, 5 and 7, Figure 1A) whereas C-terminal ligands, particularly position 12, are too distant to chelate directly (reviewed in 52). In order to catch the ion, the exiting helix (which includes position 12) must be repositioned (52,116). In ∼10% of EF-hands, position 12 is occupied by Asp instead of Glu, which reduces cation selectivity (52) (Figures 1 and 2). In the Ca^2+^-specific EF-hands, Mg^2+^ appears to bind only the ligands in the N-terminal part of the loop and this binding has no effect on the overall EF-hand conformation. In contrast, in the Ca^2+^/Mg^2+^ EF-hands the last residue can bind bidentally to Ca^2+^ but monodentally to Mg^2+^; if the site is sterically blocked into a bidentate configuration, these EF-hands become Ca^2+^-specific (117,118). Therefore, it is not inconceivable that synonymous codons at position 12 could undergo selection only in a subset of EF-hands although it is unclear if this would explain a preference for splice-repressive Glu codon at position 12 (Figure 1, Table 1). Thus, the hypothesis that evolution of EF-hand ESEs reflected a requirement for the sequential order of cation binding and Ca^2+^/Mg^2+^ selectivity may warrant further scrutiny. Combinations of various selective forces behind the codon usage bias in the EF-hand loops should be disentangled in more detail in future studies.

### Search for SR proteins that regulate inclusion of exons encoding CBSs in mRNA

Consensus sequences for EF-hand loops suggest that alternate GA-rich motifs could recruit preferred RBPs and their complexes (Figure 1A,2B). Many RBPs favour bipartite motifs flanked by preferred bases and many, if not most, ESEs and ESSs act through the formation of early exon definition complexes, with RBPs often acting additively to determine the extent of exon inclusion (7,110,119). Although a number of RBPs and their pre-mRNA targets involved in AS of Ca^2+^ signaling genes have been identified (120–123), complexes recruited to exonic sequences for EF-hand loops remain unknown. Studies employing CLIP often produced binding preferences different from those that do not depend on UV crosslinking (7,119).

ESEs containing GA dinucleotides may bind a subset of SR proteins (Supplementary Table S2) but individual GAY-to-CAC mutations and ESE-to-ESS remodeling of the DADLD site were insufficient to alter exon 4b inclusion levels by SR protein regulators (Figure 5H). The failure to respond to mutations is consistent with a significant ESE-independent component of SRSF1 action, as proposed (124,125). In the most recent model (125), SRSF1 could be directly recruited to 5′ss through interactions with stem–loop 3 of U1 small nuclear ribonucleoprotein (snRNP), namely, between SRSF1 RRM1 and the CA motif at the 5′ part of the stem-loop, and between RRM2 and a double-stranded GGA motif, with ESEs showing only low transient SRSF1 occupancy (125,126). In other words, the ESE-independent SRSF1 interactions appearred to be sufficient for connecting U1 and U2 snRNPs (125).

Optimal binding motifs for SRSF1, including GGAAGAAG (127) or UCAGAGGA (74), are purine-rich, contain GA dinucleotides (underlined) and support optimal binding of SRSF1 to GAs separated by a single purine. The AGAAGA 6-mer confers the strongest exon inclusion among all 4,096 hexamers (6). GA dinucleotides at coordinating residues of EF-hand loops are, however, typically separated by 4 nts, except for codons 11 and 12 (Figure 1). In our sample (Dataset S1), GARGA motifs were present only in ∼25% canonical EF-hand loops, with the vast majority involving codons 11 and 12 where they were ∼2.5-times more common than their GAYGA counterparts. In SRSF1-responsive *OGDH* exons 4a and 4b, the GARGA motifs are absent, but duplicated exon 4a, which lacks SRSF1 eCLIP signals, lost a half of GA dinucleotides from its ancestral copy (Figure 5G). The maximum SRSF1 eCLIP signal was at 5′ss of exon 4b in most replicas, but also at the DADLD motif (Figure 5G), raising speculation that SRSF1 interactions at ESEs and at 5′ss might reflect successive stages of kinetic proofreading (128). The eCLIP signal for SRSF1 at or close to authentic 5′ss and proximal to the SRSF1-activated cryptic 5′ss (Figure 5C–F) is in agreement with position-dependent activities of SRSF1 and other SR proteins to promote interaction of U1 with the 5′ss (129,130). The spatial separation of GA dinucleotides in codons for acidic residues in EF-hands may reduce SRSF1 binding and require action of more activator proteins, as proposed for other splicing substrates (73,74,131–133). Such cooperative action may be altered by overexpressing only one SR protein, resulting in a failure to reject cryptic sites and SR-specific cryptic splice-site patterns (Figure 5C-E) although secondary effects of the SR protein imbalance on other splicing factors cannot be excluded. The Srsf1/Srsf2 binding signatures in mice could not explain splicing activation or repression, nevertheless loss of one SR protein was accompanied by coordinated loss or compensatory gain of other SR proteins (73). In *Drosophila*, CLIP binding densities for Sf2 (SRSF1 orthologue) and other SR proteins were higher near the distal 5′ss when the SR proteins activated its usage, and higher near the proximal 5′ss when this site was promoted (134), in line with the observed splicing and binding patterns in *OGDH* (Figure 5) and with SRSF1 binding close to 5′ss generally promoting exon inclusion (74,134).

GAR triplets are also preferred binding sites for Tra2β (135,136). Individual knockdowns of either Tra2β or Tra2α did not lead to detectable changes in *OGDH* exon 4a/4b ratios (Figure 5A), with each exon containing only one GAR triplet. In the gene for calcitonin, the GAA elements in *CALCA* exon 4 were bound by protein complexes containing Tra2 and splicing activator(s) that, in turn, recruit(s) additional SR proteins (133), similar to a Tra2-activation in *Drosophila doublesex* (137). However, efficient binding to GAR motifs may require stabilization of secondary RNA structures that contain additional sites (138), which may not be accessible in a base-paired conformation (Figure 4). A similar constraint may exist for the predicted unpaired status of the codon 1 wobble (Figure 4), which should be confirmed by structural RNA probing focused on the CBP subfamilies.

### SRSF1 and Ca^2+^-binding proteins

Homozygous deletions of Srsf1 and other SR proteins lead to embryonic lethality, but conditional Srsf1 targeting implicated this protein as a key component of heart remodeling through a defect in AS of Ca^2+^/calmodulin-dependent kinase IIδ (139). Cardiomyocytes deficient in Srsf1 showed a hypercontraction defects that were associated with postnatal impairment of heart-specific AS, such as developmental switch in AS of cardiac troponin (139). Although strictly post-splicing activities of Srsf1 in the cytoplasm are dispensable for embryonic development, mice expressing Srsf1 exclusively in the nucleus display motility defects involving multiple cilia and sperm flagella (140), but Ca^2+^ signaling pathways in these animals have not been studied either. Srsf1 deletion in myogenic progenitors led to defects in neuromuscular junctions, muscle weakness and atrophy, thin myofibers, as well as exon skipping of multiple genes (141). Transcripts affected by exon skipping included genes involved in Ca^2+^ metabolism, as exemplified by kinesins, *Homer1*, integrin 7a, calpain 3 and a cholinergic receptor subunit *Chrne* (141), but the specific role of CBPs in the phenotype has not been addressed. CLIP binding sites for SRSF1 were also found in transcripts involved in Ca^2+^ metabolism, such as calmodulin and autophagy regulator beclin (72). Thus, defining the exact role of SRSF1 in regulating exons for CBSs warrants further studies.

### SND1 and LIN28B as putative regulators of EF-hand loop exons

eCLIP data revealed a preference of LIN28B and SND1 binding sites to exonic segments encoding EF-hand loops (Figure 6, Dataset S2). Both proteins are *bona fide* RBPs (76,77). A highly similar paralogue of LIN28B, LIN28A, binds mRNA motifs containing tandem GAs enriched in exons and 3′ untranslated regions and its exogenous expression led to widespread aberrant splicing (77). GA-rich motifs, including GNGAY and GGAG oligomers, are prominent at the centre of LIN28 targets and are preferentially single-stranded (77). In addition, both LIN28A and LIN28B bind and regulate a number of splicing factors, including core U1 and U2 snRNP components, with synergistic or antagonistic effects on targets in common (77).

SND1 is a multifunction protein found in higher and lower eukaryotes, including ciliates, but not in bacteria (98). Tandem SN domains in SND1 show significant sequence identity to the Ca^2+^-dependent staphylococcal nuclease, but highly conserved Asp residues involved in Ca^2+^ binding in bacteria are absent in eukaryotic SND1 orthologues (142). SND1 was shown to stimulate the first splicing step and interact with multiple snRNP components, including symmetrically dimethylarginine-modified Sm proteins (143,144). Although SND1 was not detected in proteomic analyses of spliceosomal complexes (145,146), large SND1-interacting proteins such as U5 snRNP p220 (Prp8 in yeast) (143) required fragmentation, which may lead to false negatives (146). The eCLIP signals across EF-hand loop exons could also reflect SND1 interactions with stress granules components, such as TIA-1 or TIAR (98 and refs. therein), stabilizing a subset of mRNAs. SND1 knockdown was associated with downregulation of the EF-hand protein S100A11 (147), but SND1 eCLIP signals were absent for S100 EF-hands in our sample, except for S100A1.

SND1 was reported to bind signatures of N^6^-methyladenosine (m^6^A) sites, with optimum binding to GAC-containing motifs (76). SND1 belongs to the Tudor domain ‘Royal family’ that reads methylated residues through the use of an aromatic cage (148), a structure similar to that found in YTH readers that recognize m^6^A, the most prevalent modification of eukaryotic mRNA (149). m^6^A can destabilize RNA secondary structure (150), which may indirectly recruit splicing regulators to purine-rich regions, such as one-RRM hnRNP G, alter RNA polymerase II occupancy, and in turn, splicing patterns (151–153). High-affinity interactions between hnRNP A2 and GA-rich RNA motifs were Ca^2+^-dependent in a narrow concentration range (154). Our inspection of 178 049 putative human m^6^A sites, the largest m^6^A collection to date (41), revealed GAC codons for Ca^2+^-coordinating residues that were methylated, including the fifth residue of the DADLD motif (Figure 6G), but only little overlap with the EF-hand loop codons. Because high-throughput m^6^A data suffer from substantive background signals and false positivity, we examined recent high-confidence m^6^As in miCLIP2 datasets (42). Although the overlap between predicted 36 556 m^6^A sites in 7552 genes (42) and our sample of EF-hand loops was not greater than expected (obs. 8 versus exp. 5.6 loops with m^6^A codons, *P* = 0.2, Supplementary Table S6), preferred m^6^A pentamer targets were somewhat depleted in the loops as compared to control exons (Supplementary Table S7). In our sample, the optimal m^6^A target (GGACU) was mainly found at EF-hand loop codon 9 where we found no modification. Finally, m^6^A peaks were reported to overlap with binding sites for RBM15, which forms a part of the m^6^A writer complex (155), interacts with splicing factor SF3B1 and binds transcripts involved in Ca^2+^ transport (156), but RBM15 binding to exonic regions of EF-hand loops was not enriched (Dataset S2).

### ESE-supported protein domains

High proportions of negatively charged amino acids are present in other domains, most notably ligand-binding domains of calcium-dependent endocytic receptors (49). The class A low-density lipoprotein receptor family contains up to 20% of Asp residues (49). Higher than average Asp or Glu fractions are also present in a large subset of epidermal growth factor-like modules, GoLoco motifs, KRAB/SCAN and Olduvai (also known as NBPF or DUF1220) domains as well as in other motifs with yet unknown function (49). For example, the Glu-rich Olduvai domains are built of two exons that are supported by high ESE/ESSseq profiles near splice sites and by a flanking repeat containing a series of Glu and Asp codons to enhance the use of upstream 3′ss (Supplementary Figure S9A, B). The remarkably strong inclusion capacity of the Olduvai exon doublet may have supported their spread during primate evolution. Olduvai domains show signs of positive selection, a most striking expansion of their copy number in humans and a high neuron-specific expression in cognitive regions of the brain (157,158), the tissue with the highest level of AS (159).

### Medical significance of ESEs that encode Ca^2+^-binding sites

Our data imply that exonic mutations in EF-hand loops and other CBSs carry a higher than average chance to reduce exon inclusion in mature transcripts. Disease-causing missense mutations have been reported to cluster in Ca^2+^-binding residues (160,161) and Asp to His substitutions in the epidermal growth factor-like domain can reduce exon inclusion (162). Thus, missense mutations affecting acidic residues and/or CBSs should be examined for aberrant splicing, particularly those in weak exons where the ESE/ESS enrichment/depletion is lower than in constitutive exons (6). The enhanced likelihood of pathogenic mutations in CBSs to affect pre-mRNA splicing may expand phenotypic diversity and increase disease severity of their carriers, most often through exon skipping. Finally, incorporation of metal binding residues and the I-W affinity data in variant prediction algorithms should improve their suboptimal accuracy.

## Supplementary Material

gkac270_Supplemental_Files
