# Supplementary material for: Exonic splicing code and protein binding sites for calcium
Source: Nucleic Acids Res. 2022 Apr 26;50(10):5493–512. doi: 10.1093/nar/gkac270 (PMC9177970; doi:10.1093/nar/gkac270)
Supplement: gkac270_Supplemental_Files [file gkac270_supplemental_files.zip › Captions for Datasets S1-S3.docx]

**Supplemental Datasets S1-S3 (excel files)**

**Dataset S1 List of CBPs included in the study**

**Dataset S2 Candidate RBPs identified by GAT as preferentially binding to canonical EF-hand loops**

**Dataset S3 Codon usage bias in canonical EF-hand loops**
