# Supplementary material for: Exonic splicing code and protein binding sites for calcium
Source: Nucleic Acids Res. 2022 Apr 26;50(10):5493–512. doi: 10.1093/nar/gkac270 (PMC9177970; doi:10.1093/nar/gkac270)
Supplement: gkac270_Supplemental_Files [file gkac270_supplemental_files.zip › MBS - supplemental information REVISED.pdf]

SUPPLEMENTAL INFORMATION

Exonic splicing code and protein binding sites for calcium

SUPPLEMENTAL FIGURES

Figure S1 ESEc/ESSc scores for EF-hand loop codons
A, Canonical EF-hands. **B,** PseudoEF-hands in S100 proteins. **C,** other noncanonical EF-hands. Average ESEc/ESSc scores for protein-coding human exons are denoted by dotted lines.

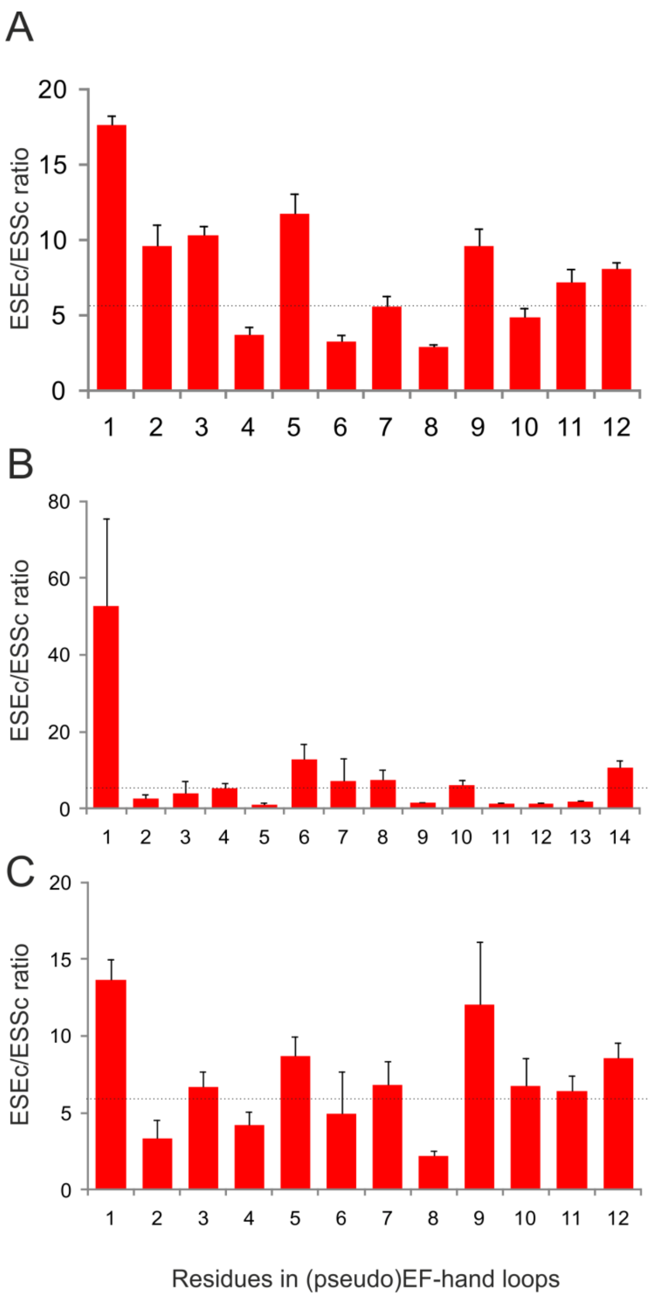

Figure S2 Codon usage in canonical EF-hand loops, their splicing potential and cell differentiation

A, Relative synonymous codon usage and *ln*(ESEf/ESSf) scores. **B,** Comparison of codon usage frequencies at loop positions 1 and 12 with codon usage frequencies in 92 genes active in the M phase of mitotic cell cycle (‘proliferation’, *blue* columns) and 82 genes involved in pattern specification (‘differentiation’, *red* columns). Red and blue data were taken from Gingold et al. (1) and green data from Dataset S3. **C,** Codon usage signatures in genes involved in proliferation or differentiation and predicted splicing activities of all synonymous codons. Each red diamond represents one synonymous codon of the standard genetic code (n=59, excluding Trp, Met and stop codons).Y-axis values show fold excess of codon usage frequencies in genes involved in pattern specification over mitotic activity (1). Dotted line divides codon usage signatures in genes involved in proliferation (<1, *grey ovals*) and differentiation (>1, *green and yellow ovals*). The yellow oval contains rare codons: they are enriched in the differentiation set and have usage frequencies in genes involved in mitotic activity <0.12 (1). Note that preferred codons at EF-hand loop positions 1 and 12 are in the green oval.

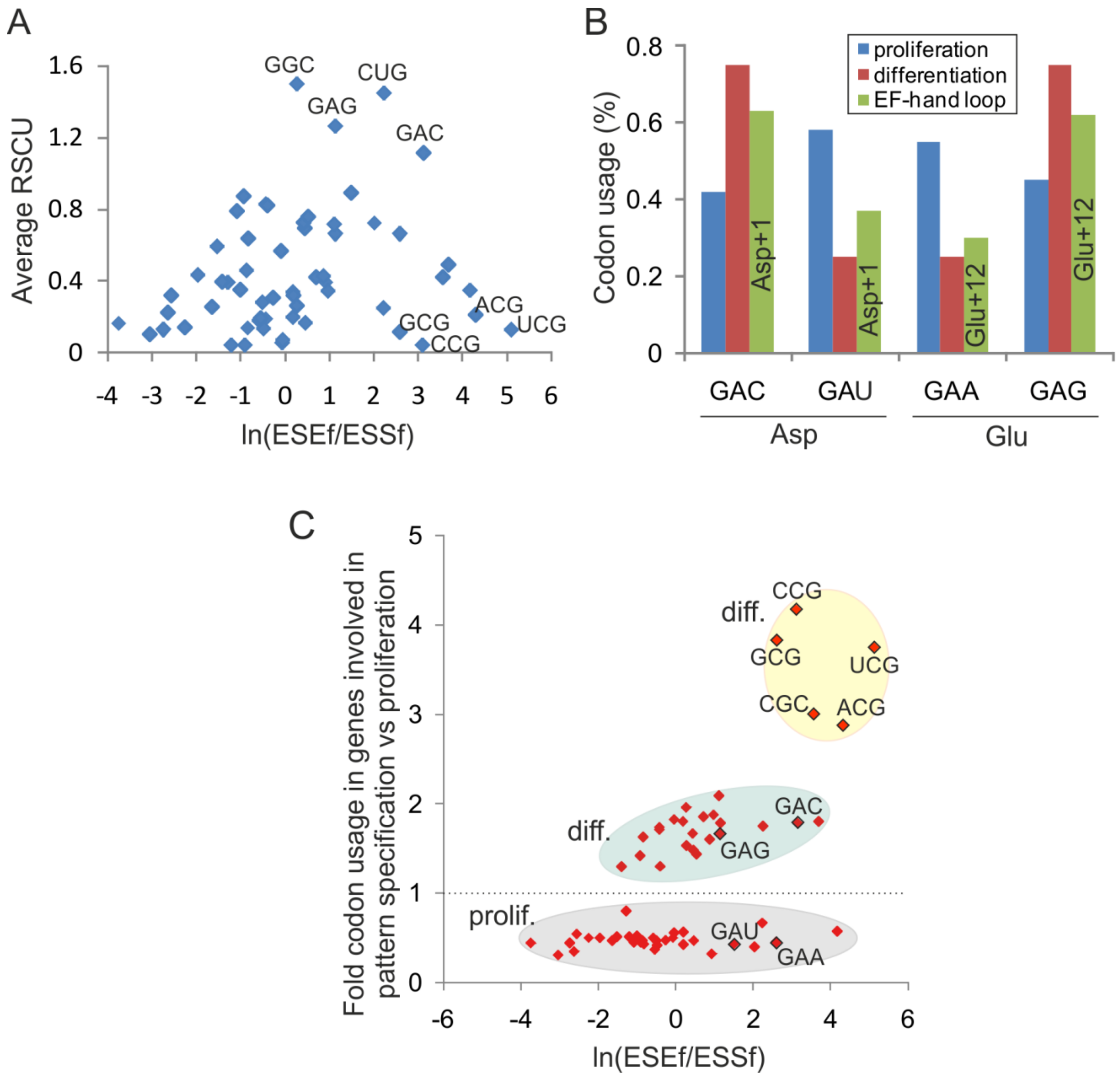

Figure S3 Traditional splicing motifs that flank exons encoding Ca^{2+}-binding sites
A, Ca^{2+}-binding motifs encoded by a single exon. **B,** Ca^{2+}-binding motifs encoded by 2 or more exons. **C, D,** Mean distances between predicted branch points with the highest SVM-BP scores and 3' splice site. Error bars are SDs. **E,** The intrinsic strength of 3' and 5' splice sites of exons encoding the indicated EF-hands, expressed as maximum entropy (maxent) scores (2). Error bars are SDs. The strength of 5'ss showed weak negative correlation with average ESE/ESSseq scores of canonical EF-hand motifs (r=-0.14, *P*=0.03).

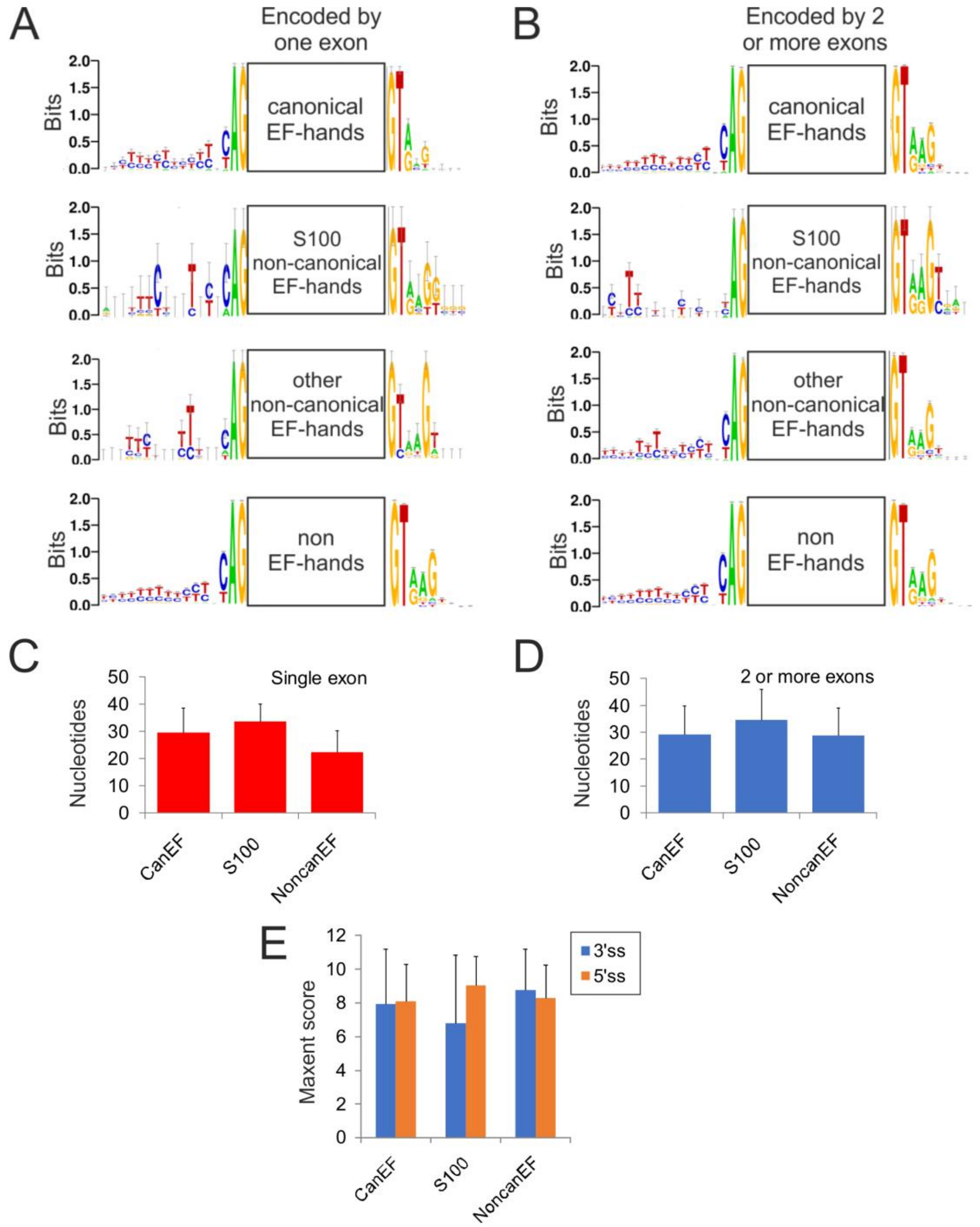

Figure S4 Nucleotide frequencies in exonic segments encoding split EF-hand loops
A, Canonical EF-hand loops. **B,** Noncanonical EF-hand loops**.**

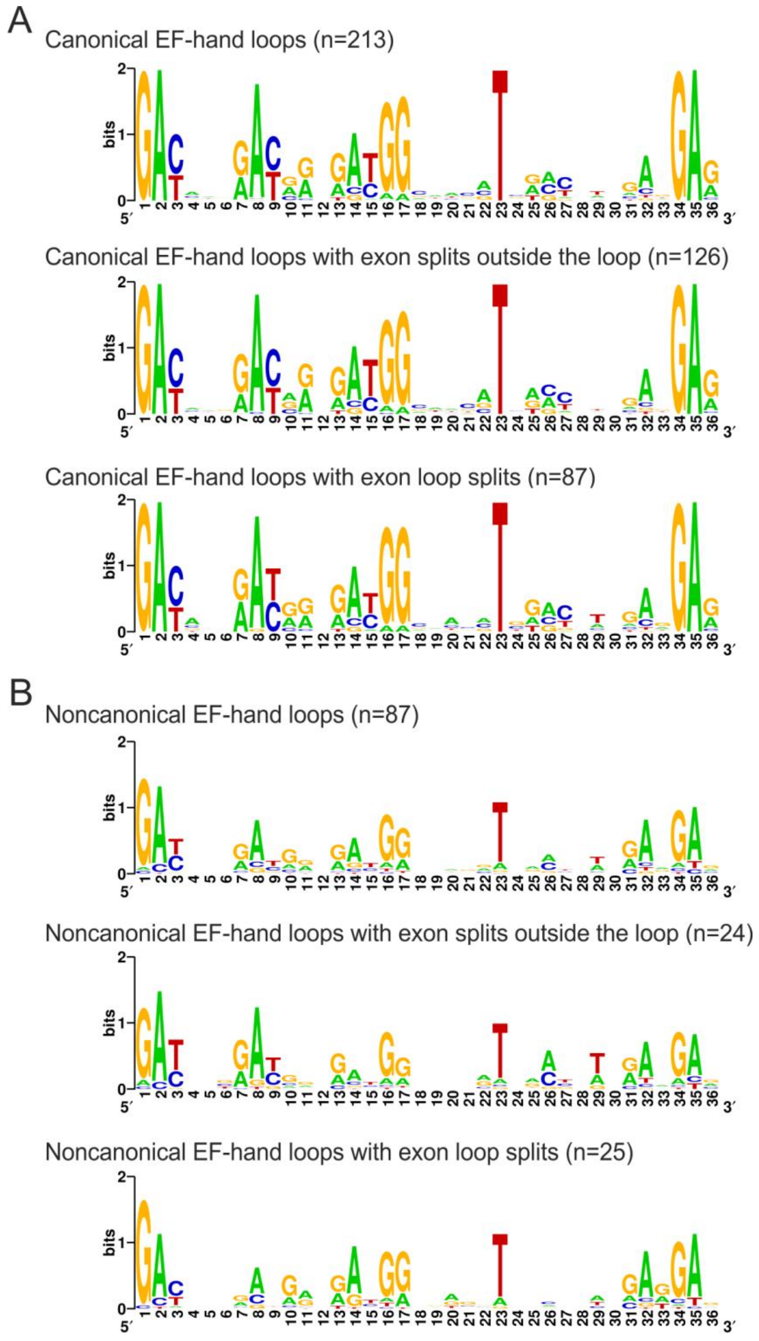

Figure S5 ESE/ESSseq scores for EF-hand motifs encoded by one *vs.* two or more exons
A, Canonical EF-hands. **B,** Noncanonical EF-hands**.** Grey rectangles denote nucleotides that code for the Ca^{2+}-binding loop.

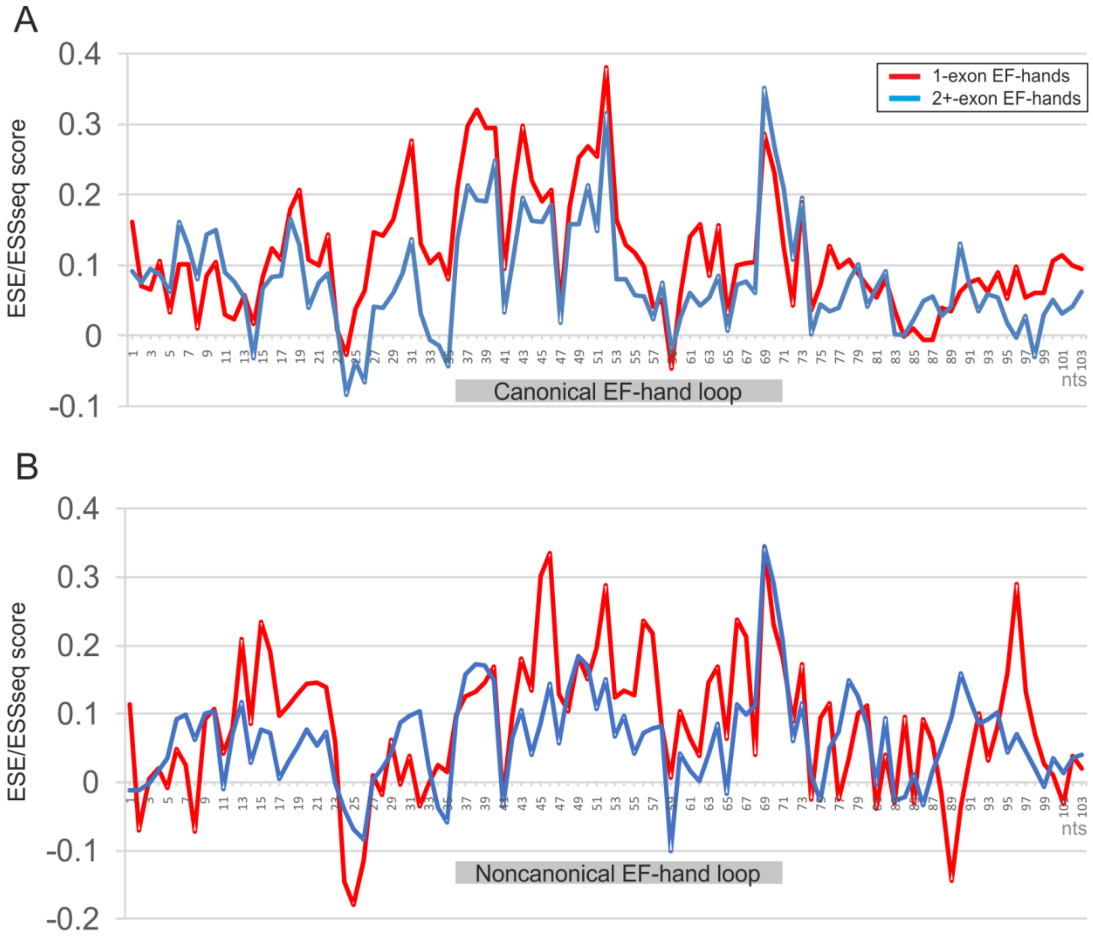

Figure S6 GA-containing motifs in high-confidence ESEs and ESSs
A, Proportion of the indicated dinucleotides in 1,182 ESE and 1,090 ESS hexamers identified previously (3). **B,** Enrichment of the indicated dinucleotides in high-confidence 1,182 ESEs over 1,090 ESSs. Dotted line denotes equal dinucleotide proportions in ESEs and ESSs. **C,** Fractions of GAN-containing hexamers in the same ESE/ESS collection.

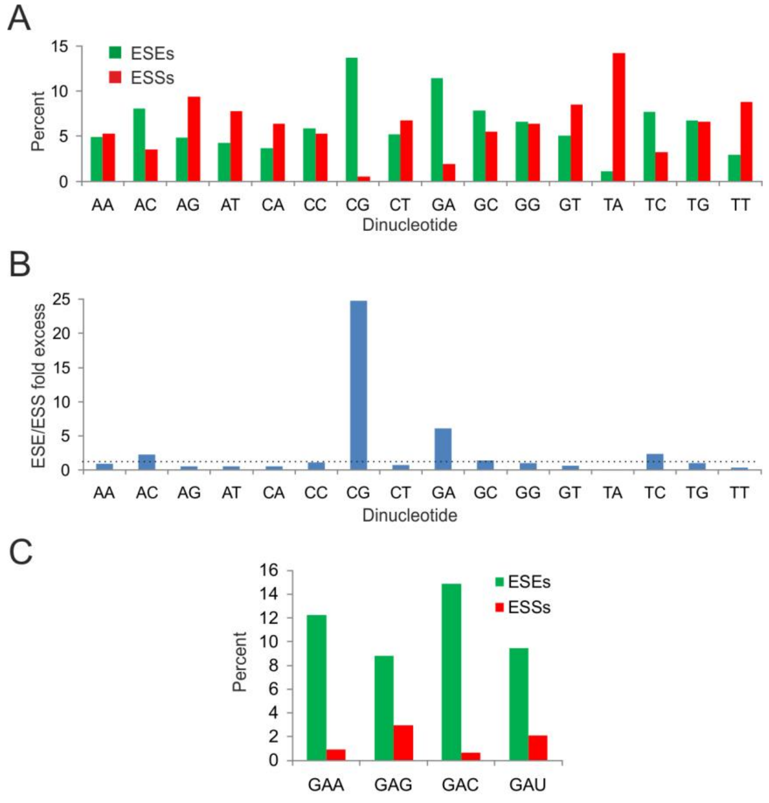

Figure S7 mRNA base-pairing probabilities across the EF-hand loop of the indicated CBPs
Nucleotides encoding the 12-amino acid EF-hand loop are numbered from the first position of the first codon. BPP, cumulative base-pairing probabilities, S, enthropy. Red rectangles denote codons for Ca^{2+}-coordinating amino acids. Number of EF-hand loops in core family is in parentheses.

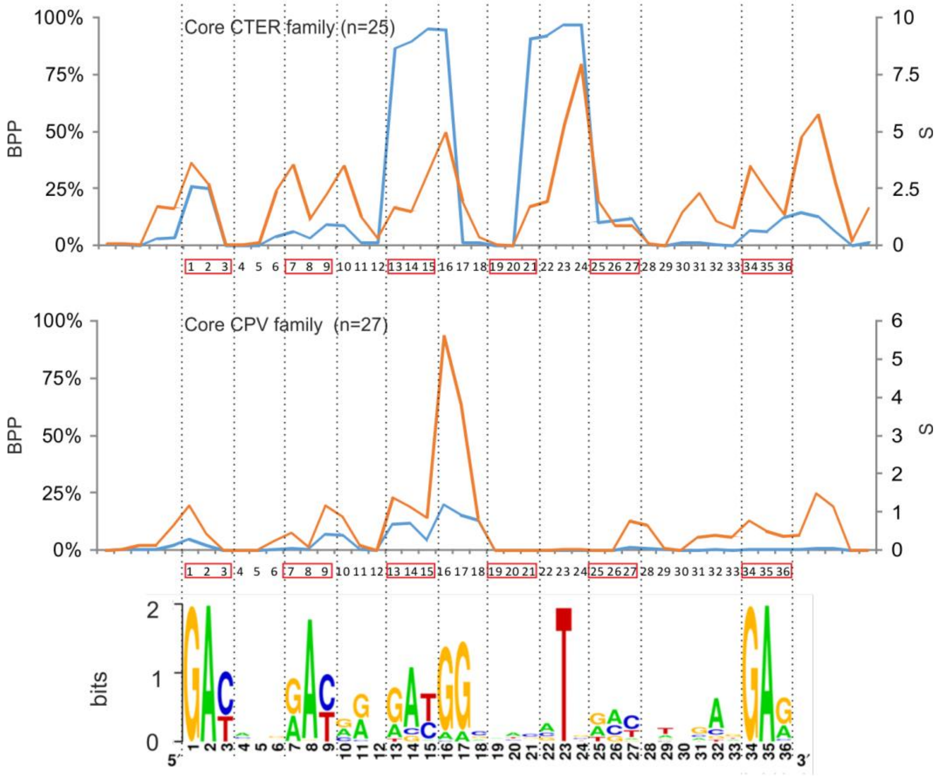

Figure S8 Factors that may influence codon usage at position 6 of canonical EF-hand loops
A, Usage of Gly codons (*columns*) and their predicted exon inclusion potential (*red markers*). F, fragile codon (converted to nonsense codon by a single point mutation), R, robust codons (require at least 2 point mutations to nonsense) (4). **B,** Fold difference in Gly codon usage in 82 genes involved in patterning specification over 92 genes involved in mitotic cell division. Codon frequences were taken from Gingold et al. (1).

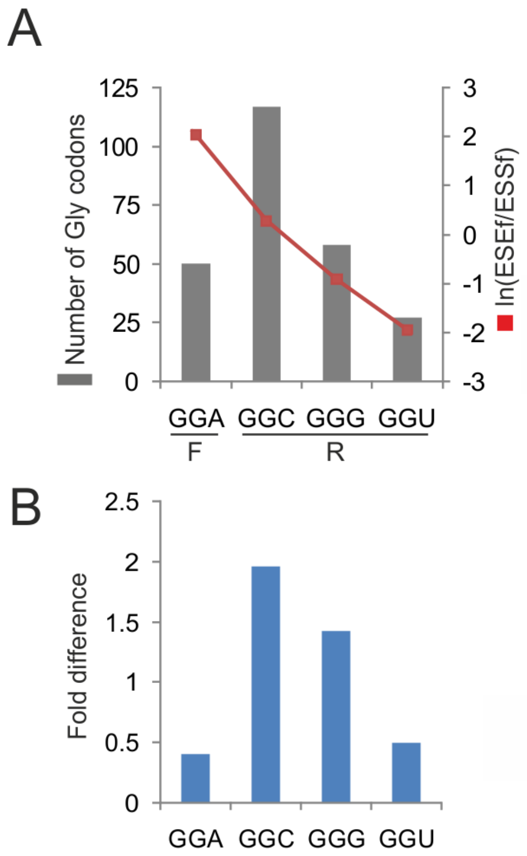

Figure S9 ESE/ESS profiles for exon doublets that encode Olduvai domains in *NBPFx* genes
A, Average ESE/ESSseq scores across the two exons (numbered here I and II). Olduvai (also known as NBPF or DUF1220) domain is in *green*. A flanking Glu- and Asp-rich segment, potentially derived from a repeat, is in *blue*. *Red* circles denote acidic residues. Both exons are aligned with intervening introns in panel B. **B,** Multiple alignment of corresponding Olduvai-encoding gene segments. Exons I and II are in upper case, flanking introns are in lower case. Exonic parts encoding the Olduvai domains are in *green*, as delineated previously (5). Amino acids flanking the splice sites of the intervening intron are shown for *NBPF3*. The 5' part of the first exon (*blue*) resembles simple $(AGA)_n$ or $(GATGAA)_n$ repeats detected by RepeatMasker (v. 4.0.9). A large intron insertion specific for *NBPF4*, *NBPF6* and *NBPF8* shows 94% identity to a chimpanzee orthologue and 72% identity to a macaque orthologue (default megablast options). Predicted branch point adenosines are in yellow.

A

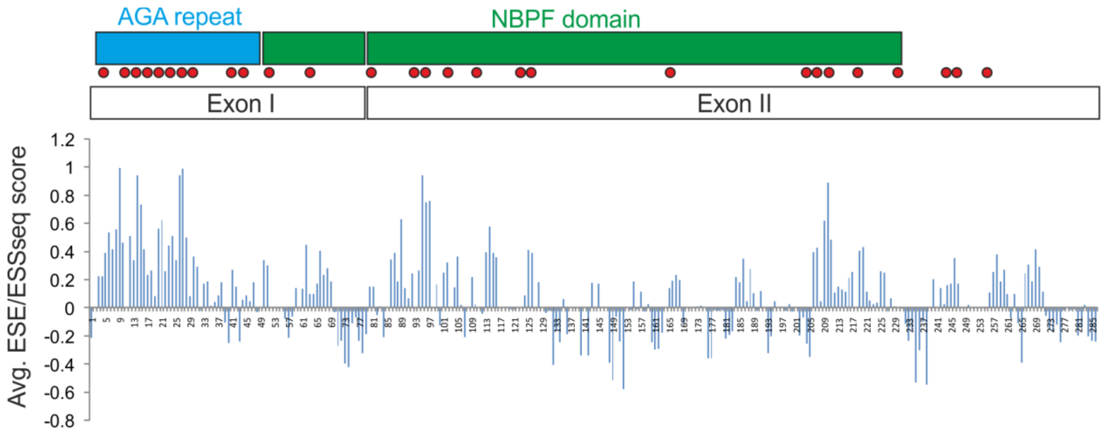

B

```
NBPF3           gaaatatctgaatgaacacttctgtatttacagAAAATGATGACGA------TGAGGATG
NBPF1-1         gaaatatctgaacgaacatt-ttgtatttacagAAAATGACGAAGA------TGAGGATG
NBPF1-2         gaaatatctgaacgaacatt-ttgtatttacagAAAATGACGAAGA------TGAGGATG
NBPF15          gaaatatctgaacgaacacttctgtatttacagAAAATGACAACGA------TGACGATG
NBPF14-1        gaaatatctgaacgaacatt-ttgtatttctagAAAATGACGAAGA------TGAGGATG
NBPF14-2        gaaatacctgaacgaacacttctgtatttacagAAAATGACAACGA------TGACGATG
NBPF20          gaaatatctgaacgaacacttctgtatttacagAAAATGACAACGA------TGACGATG
NBPF9-1         gaaatatctgaatgaacatt-ttgtatttctagAAAATGACGAAGA------TGAGGATG
NBPF9-2         gaaatatctgaacgaacacttctgtatttacagAAAATGACAACGA------TGACGATG
NBPF12          gacatatctgaatgaacatt-ttgtatttatagAAAATGATGAAGA------TGAGGATG
NBPF12-1        aaaatatctgaacgaacatt-ttgtatttatagAAAATGATGAAGA------TGAGGATG
NBPF12-2        gaaatatctgaatgaacacttctgtatttacagAAAATGATAACGA------TGACGATG
NBPF10-1        gaaatatctgaacgaacatt-ttgtatttctagAAAATGACGAAGA------TGAGGATG
NBPF10-2        gaaatacctgaacgaacacttctgtatttgcagAAAATGACAACGA------TGACGATG
NBPF11-1        gaaatatctgaacgaacatt-ttgtatttatagAAAATGACGAAGA------TGAGGATG
NBPF11-2        gaaatatctgaatgaacacttctgtatttacagAAAATGATAACGA------TGACGATG
NBPF19          gaaatatctgaacgaacacttctgtatttacagAAAATGACAACGA------TGACGATG
NBPF26-1        gaaatatctgaacgaacatt-ttgtatttatagAAAATGACGAAGA------TGAGGATG
NBPF26-2        gaaatatctgaacgaacacttctgtatttacagAAAATGACAACGA------TGACGATG
NBPF8           gaaatatttcaatggacacatctgtatttgcagAAAATGATGAAGATGAAGATGAGGATG
NBPF4           gaaatatttcaatggacacatctgtatttgcagAAAATGATGAAGATGAAGATGAGGATG
NBPF6           gaaatatttcaatggacacatctgtatttgcagAAAATGATGAAGATGAAGATGAGGATG
                 * ***  * ** * ***    *******  *********  * **      *** ****

                                         E  K  V  Q  E  L  Y  A  P  R
NBPF3           AAGATGTTAAAGTTGAGGAGGCTGAGAAAGTACAGGAATTATATGCCCCCAGgtaacgct
NBPF1-1         AAGATGTTCAAGTTGAGGAGGCTGAGAAAGTACTGGAATCATCTGCCCCCAGgtaacact
NBPF1-2         AAGATGTTCAAGTTGAGGAGGCTGAGAAAGTACTGGAATCATCTGCCCCCAGgtaacact
NBPF15          AAGATGTTCAAGTTGAGGTGGCTGAGAAAGTGCAGAAATCGTCTGCCCCCAGgtaacact
NBPF14-1        AAGATGTTCAAGTTGAGGAGGCTGAGAAAGTACTGGAATCATCTGCCCCCAGgtaacact
NBPF14-2        AAGATGTTCAAGTTGAGGTGGCTGAGAAAGTGCAGAAATCGTCTGCCCCCAGgtaacact
NBPF20          AAGATGTTCAAGTTGAGTTGGCTGAGAAAGTGCAGAAATCGTCTGCCCCCAGgtaacact
NBPF9-1         AAGATGTTCAAGTTGAGGAGGATGAGAAAGTACTGGAATCATCTGCCCCCAGgtaacact
NBPF9-2         AAGATGTTCAAATTGAGGTGGCTGAGAAAGTGCAGAAATCGTCTGCCCCCAGgtaacact
NBPF12          AAGATGTTCAAGTTGAGGAGGATGAGAAAGTACTGGAATCATCTGCCCCCAGgtaacact
NBPF12-1        AAGATGTTCAAGTTGAGGAGGATGAGAAAGTGCTGGAATCATCTTCCCCCAGgtgacact
NBPF12-2        AAGATGTTCAAGTTGAGGTGGCTGAGAAAGTGCAGAAATCGTCTTCCCCCAGgtaacact
NBPF10-1        AAGATGTTCAAGTTGAGGAGGCTGAGAAAGTACTGGAATCATCTGCCCCCAGgtaacact
NBPF10-2        AAGATGTTCAAGTTGAGGTGGCTGAGAAAGTGCAGAAATCATCTGCCCCCAGgtaacact
NBPF11-1        AAGATGTTCAAGTTGAGGAGGATGAGAAAGTACTGGAATCATCTGCCCCCAGgtaacatt
NBPF11-2        AAGATGTTCAAGTTGAGGTGGCTGAGAAAGTGCAGAAATCGTCTTCCCCCAGgtaacact
NBPF19          AAGATGTTCAAGTTGAGGTGGCTGAGAAAGTGCAGAAATCGTCTGCCCCCAGgtaacact
NBPF26-1        AAGATGTTCAAGTTGAGGAGGATGAGAAAGTACTGGAATCATCTGCCCCCAGgtaacact
NBPF26-2        AAGATGTTCAAGTTGAGGTGGCTGAGAAAGTGCAGAAATCGTCTGCCCCCAGgtaacact
NBPF8           AAGACGACAAAGACGAGGAGGTTGAGAAAGTACAGGAATCACCTGCCCCCAGgtaacatt
NBPF4           AAGACGACAAAGACGAGGAGGTTGAGAAAGTACAGGAATCACCTGCCCCCAGgtaacatt
NBPF6           AAGACGACAAAGACGAGGAGGTTGAGAAAGTACAGGAATCACCTGCCCCCAGgtaacatt
                **** *   **   ***  ** ********* * * ***    * ********* **  *

NBPF3           gaataatcgggagcaagtaatgggtggtaacatatgaaaaaggtc---------------
NBPF1-1         gaatactcgggagcaagtaatgggtggtaacatatgaa-aatgtg---------------
NBPF1-2         gaatactcaggagcaagtaatgggtggtaacatatgaa-aatgtc---------------
NBPF15          gaatactcaggaacaattaatggatggtaacatatgaggaatatc---------------
NBPF14-1        gaatactcaggagcaagtaatgggtggtaacgtatgaa-aatgtc---------------
NBPF14-2        gaatactcaggaacaattaatggatggtaacatatgaggaatatc---------------
NBPF20          gaatactcaggaacaattaatggatggtaacatatgaggaatatc---------------
NBPF9-1         gaatactcaggagcaagtaatgggtggtaacatatgaa-gatgtc---------------
NBPF9-2         gaatactcaggaacaattaatggatggtaacatatgaggaatatc---------------
NBPF12          gaatactcaggagcaagtaatgggtggtaacatatgaa-aatgtc---------------
NBPF12-1        gaatactcaggagcaagtaatgggtgttaacatatgaa-aatgtc---------------
NBPF12-2        gaatactcaggaacaattaatggatggtaacatatgaagaatatc---------------
NBPF10-1        gaatactcaggagcaagtaatgggtggtaacgtatgaa-aatgtc---------------
NBPF10-2        gaatactcaggaacaattaatggatggtaacatatgaggaatatc---------------
NBPF11-1        gaatactcaggagcaagtaatgggtggtaacatatgaa-aatgtc---------------
NBPF11-2        gaatactcaggaacaattaatggatggtaacatatgaagaatatc---------------
NBPF19          gaatactcaggaacaattaatggatggtaacatatgaggaatatc---------------
NBPF26-1        gaatactcaggagcaagtaatgggtggtaacatatgaa-aatgtc---------------
NBPF26-2        gaatactcaggaacaattaatggatggtaacatatgaggaatatc---------------
NBPF8           gaataatcaggagcgggtaatgggtggtaaaatatgaaaaaggtctcagaaagaataaaa
NBPF4           gaataatcaggagcgggtaatgggtggtaaaatatgaaaaaggtctcagaaagaataaaa
NBPF6           gaataatcaggagcgggtaatgggtggtaaaatatgaaaaaggtctcagaaagaataaaa
                ***** ** *** *   ****** ** ***  *****   *  *
```

```
NBPF3         ------------------------------------------------------------
NBPF1-1       ------------------------------------------------------------
NBPF1-2       ------------------------------------------------------------
NBPF15        ------------------------------------------------------------
NBPF14-1      ------------------------------------------------------------
NBPF14-2      ------------------------------------------------------------
NBPF20        ------------------------------------------------------------
NBPF9-1       ------------------------------------------------------------
NBPF9-2       ------------------------------------------------------------
NBPF12        ------------------------------------------------------------
NBPF12-1      ------------------------------------------------------------
NBPF12-2      ------------------------------------------------------------
NBPF10-1      ------------------------------------------------------------
NBPF10-2      ------------------------------------------------------------
NBPF11-1      ------------------------------------------------------------
NBPF11-2      ------------------------------------------------------------
NBPF19        ------------------------------------------------------------
NBPF26-1      ------------------------------------------------------------
NBPF26-2      ------------------------------------------------------------
NBPF8         gggaggtgaaggtagtcacagattccagaggcaggataaagaaagctgcagagtgcactg
NBPF4         gggaggtgaaggtagtcacagattccagaggcaggataaagaaagctgcagagtgcactg
NBPF6         gggaggtgaaggtagtcacagattccagaggcaggataaagaaagctgcagagtgcactg

NBPF3         ------------------------------------------------------------
NBPF1-1       ------------------------------------------------------------
NBPF1-2       ------------------------------------------------------------
NBPF15        ------------------------------------------------------------
NBPF14-1      ------------------------------------------------------------
NBPF14-2      ------------------------------------------------------------
NBPF20        ------------------------------------------------------------
NBPF9-1       ------------------------------------------------------------
NBPF9-2       ------------------------------------------------------------
NBPF12        ------------------------------------------------------------
NBPF12-1      ------------------------------------------------------------
NBPF12-2      ------------------------------------------------------------
NBPF10-1      ------------------------------------------------------------
NBPF10-2      ------------------------------------------------------------
NBPF11-1      ------------------------------------------------------------
NBPF11-2      ------------------------------------------------------------
NBPF19        ------------------------------------------------------------
NBPF26-1      ------------------------------------------------------------
NBPF26-2      ------------------------------------------------------------
NBPF8         atttcatgtgctcacccaacaaggaaatagccccattaacgtgcttgtccattgtcttcc
NBPF4         atttcatgtgctcacccaacaaggaaatagccccattaacgtgcttgtccattgtcttcc
NBPF6         atttcatgtgctcacccaacaaggaaatagccccattaacgtgcttgtccattgtcttcc

NBPF3         ------------------------------------------------------------
NBPF1-1       ------------------------------------------------------------
NBPF1-2       ------------------------------------------------------------
NBPF15        ------------------------------------------------------------
NBPF14-1      ------------------------------------------------------------
NBPF14-2      ------------------------------------------------------------
NBPF20        ------------------------------------------------------------
NBPF9-1       ------------------------------------------------------------
NBPF9-2       ------------------------------------------------------------
NBPF12        ------------------------------------------------------------
NBPF12-1      ------------------------------------------------------------
NBPF12-2      ------------------------------------------------------------
NBPF10-1      ------------------------------------------------------------
NBPF10-2      ------------------------------------------------------------
NBPF11-1      ------------------------------------------------------------
NBPF11-2      ------------------------------------------------------------
NBPF19        ------------------------------------------------------------
NBPF26-1      ------------------------------------------------------------
NBPF26-2      ------------------------------------------------------------
NBPF8         tggtgcctgtaagcatgaccctagatgaactcaccatccctagggacttcctgcacacac
NBPF4         tggtgcctgtaagcatgaccctagatgaactcaccatccctagggacttcctgcacacac
NBPF6         tggtgcctgtaagcatgaccctagatgaactcaccatccctagggacttcctgcacacac

NBPF3         ------------------------------------------------------------
NBPF1-1       ------------------------------------------------------------
NBPF1-2       ------------------------------------------------------------
NBPF15        ------------------------------------------------------------
NBPF14-1      ------------------------------------------------------------
NBPF14-2      ------------------------------------------------------------
NBPF20        ------------------------------------------------------------
NBPF9-1       ------------------------------------------------------------
NBPF9-2       ------------------------------------------------------------
NBPF12        ------------------------------------------------------------
NBPF12-1      ------------------------------------------------------------
NBPF12-2      ------------------------------------------------------------
NBPF10-1      ------------------------------------------------------------
NBPF10-2      ------------------------------------------------------------
NBPF11-1      ------------------------------------------------------------
NBPF11-2      ------------------------------------------------------------
NBPF19        ------------------------------------------------------------
NBPF26-1      ------------------------------------------------------------
NBPF26-2      ------------------------------------------------------------
NBPF8         agaggagacctgttctcccctgtagtgaaagccaggatgagatgtaaagctgctttctac
NBPF4         agaggagacctgttctcccctgtagtgaaagccaggatgagatgtaaagctgctttctac
NBPF6         agaggagacctgttctcccctgtagtgaaagccaggatgagatgtaaagctgctttctac
```

```
NBPF3         ------------------------------------------------------------
NBPF1-1       ------------------------------------------------------------
NBPF1-2       ------------------------------------------------------------
NBPF15        ------------------------------------------------------------
NBPF14-1      ------------------------------------------------------------
NBPF14-2      ------------------------------------------------------------
NBPF20        ------------------------------------------------------------
NBPF9-1       ------------------------------------------------------------
NBPF9-2       ------------------------------------------------------------
NBPF12        ------------------------------------------------------------
NBPF12-1      ------------------------------------------------------------
NBPF12-2      ------------------------------------------------------------
NBPF10-1      ------------------------------------------------------------
NBPF10-2      ------------------------------------------------------------
NBPF11-1      ------------------------------------------------------------
NBPF11-2      ------------------------------------------------------------
NBPF19        ------------------------------------------------------------
NBPF26-1      ------------------------------------------------------------
NBPF26-2      ------------------------------------------------------------
NBPF8         actgttgtctttaggttcttttttaggaaagataaatagcagagaggcaacaagcagagga
NBPF4         actgttgtctttaggttcttttttaggaaagataaatagcagagaggcaacaagcagagga
NBPF6         actgttgtctttaggttcttttttaggaaagataaatagcagagaggcaacaagcagagga

NBPF3         ------------------------------------------------------------
NBPF1-1       ------------------------------------------------------------
NBPF1-2       ------------------------------------------------------------
NBPF15        ------------------------------------------------------------
NBPF14-1      ------------------------------------------------------------
NBPF14-2      ------------------------------------------------------------
NBPF20        ------------------------------------------------------------
NBPF9-1       ------------------------------------------------------------
NBPF9-2       ------------------------------------------------------------
NBPF12        ------------------------------------------------------------
NBPF12-1      ------------------------------------------------------------
NBPF12-2      ------------------------------------------------------------
NBPF10-1      ------------------------------------------------------------
NBPF10-2      ------------------------------------------------------------
NBPF11-1      ------------------------------------------------------------
NBPF11-2      ------------------------------------------------------------
NBPF19        ------------------------------------------------------------
NBPF26-1      ------------------------------------------------------------
NBPF26-2      ------------------------------------------------------------
NBPF8         aataggaagcacctagcaaagttgaacacaaagtacagtacctagacaaaaaaatttaca
NBPF4         aataggaagcacctagcaaagttgaacacaaagtacagtacctagacaaaaaaatttaca
NBPF6         aataggaagcacctagcaaagttgaacacaaagtacagtacctagacaaaaaaatttaca

NBPF3         ---------------------------------taggaggcacaccctctctggcatcta
NBPF1-1       ---------------------------------taggaggcacaccctctctggcatcta
NBPF1-2       ---------------------------------taggaggcacaccctctctggcatcta
NBPF15        ---------------------------------taggaggcacaccctctctggcatcta
NBPF14-1      ---------------------------------taggaggcacaccctctctggcatcta
NBPF14-2      ---------------------------------taggaggcacaccctctctggcatcta
NBPF20        ---------------------------------taggaggcacaccctctctggcatcta
NBPF9-1       ---------------------------------taggaggcacaccctctctggcatcta
NBPF9-2       ---------------------------------taggaggcacaccctctctggcatcta
NBPF12        ---------------------------------tagaaggcacaccctctctggcatcta
NBPF12-1      ---------------------------------taggaggcatgccctctctggcatcta
NBPF12-2      ---------------------------------taggaggcacaccctctctggcatgta
NBPF10-1      ---------------------------------taggaggcacaccctctctggcatcta
NBPF10-2      ---------------------------------taggaggcacaccctctctggcatcta
NBPF11-1      ---------------------------------taggaggcacgccctctctggcatcta
NBPF11-2      ---------------------------------taggaggcacaccctctctggcatgta
NBPF19        ---------------------------------taggaggcacaccctctctggcatcta
NBPF26-1      ---------------------------------taggaggcacaccctctctggcatcta
NBPF26-2      ---------------------------------taggaggcacaccctctctggcatcta
NBPF8         tttcatgtcacaatattaaaattttaaaaaaactagaaggcacaccatctctgaagtcta
NBPF4         tttcatgtcacaatattaaaattttaaaaaaactagaaggcacaccatctctgaagtcta
NBPF6         tttcatgtcacaatattaaaattttaaaaaaactagaaggcacaccatctctgaagtcta
                                              *** *****  ** ******   * **
```

```
NBPF3          tgatgggccgaaagcctgcattcccttggccacagtatgagaaactcaagccagcttgga
NBPF1-1        tgatgggccaaaagcccgcattcgcttggccacagtatgtgaaattcaacccagcttaga
NBPF1-2        tgatgggccaaaagcccgcattcgcttggccacagtatgtgaaattcaacccagcttaga
NBPF15         tgatgggccaaaaacccgcattcgcttggccacagtatgtgaaatataacccagcttaga
NBPF14-1       tgatgggccaaatgcccgcattcccttggccacagtatgtgaaattcaacccagcttaga
NBPF14-2       tgatgggccaaaaacccgcattcgcttggccacagtatgtgaaatataatccagcttaga
NBPF20         tgatcggccaaaaacccacatttgcttggccacagtatgtgaaatataacccagcttaga
NBPF9-1        tggtgggccaaaagcccgcatcccttggccacagtatgtgaaattgaacccagcttaga
NBPF9-2        tgatgggccaaaaacccgcattcgcttggccacagtatgtgaaattgaacccagcttaga
NBPF12         tggtgggccaaaagcccgcattcccttggccacagtatgtgaaattcaacccagcttaga
NBPF12-1       tgatgggccaaaagcccgcattcccttggccacagtatgtgaaattcaacccagcttaga
NBPF12-2       tgatgggccaaaaacccgcattcgcttggccacagtatgtgaaatataacccagcttaga
NBPF10-1       tgatgggccaaatgcccgcattcccttggccacagtatgtgaaattgaacccagcttaga
NBPF10-2       tgatgggccaaaaacccgcattcgcttggccacagtatgtgaaatataatccagcttaga
NBPF11-1       tgatgggccaaaagcccgcattcccttggccacagtatgtgaaattcaacccagcttaga
NBPF11-2       tgatgggccaaaaacccgcattcgcttggccacagtatgtgaaatataacccagcttaga
NBPF19         tgatgggccaaaaacccgcattcgcttggccacagtatgtgaaatataatccagcttaga
NBPF26-1       tggtgggccaaaagcccacatccccttggccacagtatgtgaaattcaacccagcttaga
NBPF26-2       tgatggaccaaaaacccgcattcgcttggccacagtatgtgaaatataacccagcttaga
NBPF8          caatggctcaaatgcctgtatagccatggccacagtatgttaactacaacccagcttaga
NBPF4          caatggctcaaatgcctgtatagccatggccacagtatgttaactacaacccagcttaga
NBPF6          caatggctcaaatgcctgtatagccatggccacagtatgttaactacaacccagcttaga
                  * *  * **  **   **   * *************  **    ** ******* **

NBPF3          cacagggtgtggcagctgtt-gtgtttctctgtgtgtggtgggtgtcatgtctgtaccgt
NBPF1-1        cacagggtgcggcagctgtc-gtgtttctctatgtgtgccaagtgtcatgtctgtaccat
NBPF1-2        cacagggtgtggcagctgtc-gtgtttctctatgtgtgccaagtgtcatgtctgtaccat
NBPF15         cacagggtgcggcagctgtc-atgtttctctgtgtgtgccgagtgtcatgtctgcaccgt
NBPF14-1       cacagggtgcggcagctgtc-gtgtttctctatgtgtgccaagtgtcatgtctgtaccat
NBPF14-2       cacagggtgcggtagctgtc-atgtttctctgtgtgtgctgagtgtcatgtctgcaccgt
NBPF20         cacagggtgcggcagctgtc-atgtttctctgtgtgtgccgagtgtcacgtctgcaccgt
NBPF9-1        cacagggtgcggcagctgtc-gtgtttctctatgtgtgccaagtgtcatgtctgtaccat
NBPF9-2        cacagggtgcggcagctgtc-atgtttctctatgtgtgccgagtgtcatgtctgcaccgt
NBPF12         cacagggtgcgacagctgtc-atgtttctctatgtgtgccaagtgtcatgtctgtaccat
NBPF12-1       cacagggtgtggcagctgtc-gtgtttctgtatgtgtgccaagtgtcatgtctgtaccat
NBPF12-2       cacagggtgcggcaggtgtc-atgtttctctatgtgtgccgagtgtcatgtctgcaccgt
NBPF10-1       cacagggtgcggcagctgtc-gtgtttctctatgtgtgccaagtgtcatgtctgtaccat
NBPF10-2       cacagggtgcggtagctgtc-atgtttctctatgtgtgctgagtgtcatgtctgcaccgt
NBPF11-1       cacagggtgcggcagctgtt-gtgtttctctatgtgtgccaagtgtcatgtctgtaccat
NBPF11-2       cacagggtgcggcagctgtc-atgtttctctatgtgtgccgagtgtcatgtctgcaccgt
NBPF19         cacagggtgcggtagctgtc-atgtttctctatgtgtgctgagtgtcatgtctgcacctt
NBPF26-1       cacagggtgcggcagctgtc-gtgtttctctatgtgtgccaagtgtcatgtctgtaccat
NBPF26-2       cacagggtgcggtagctgtc-atgtttctctatgtgtgccgagtgtcatgtctgcaccgt
NBPF8          cacaccatgtggcagctgtttttggttctctgtgtgtgc----tgtcaagactgtaccat
NBPF4          cacaccatgtggcagctgtttttggttctctgtgtgtgc----tgtcaagactgtaccat
NBPF6          cacaccatgtggcagctgtttttggttctctgtgtgtgc----tgtcaagactgtaccat
               ****   ** *  ** ***   ** **** * ******     ***** * *** *** *

NBPF3          acagggatagctgagttttcatcctcctcggctcctatctgtccagtgcaatgaacacca
NBPF1-1        acagggatagctgagtcttcatcctcctcagctcctatctgtccagtgcactgaacacca
NBPF1-2        acagggatagctgagtcttcatcctcctcagctcctatctgtccagtgcactgaacacca
NBPF15         acagggatagctgagtcttcatcctcctcagctcctatctgtccagtgcaatgaacagca
NBPF14-1       acagggatagctgagtcttcatcctcctcagctcctatctgttcagtgcactgaacacca
NBPF14-2       acagggatagctgagtcttcatcctcctcagctcctatctgtccagtgcaatgaacagca
NBPF20         acagggatagctgagtcttcatcctcctcagctcctatctgtccagtgcaatgaacagca
NBPF9-1        acagggatagctgagtcttcatcctcctcagctcctatctgtccagtgcactgaacacca
NBPF9-2        acaaggatagctgagtcttcatcctcctcagctcctatctgtccagtgcaatgaacagca
NBPF12         acagggatagctgagtcttcatcctcctcagctcctatctgtccagtgcactgaacacca
NBPF12-1       acagggatagctgagtcttcatcctcctcagctcctatttgtccagtgcactgaacacca
NBPF12-2       acagggatagctgagtcttcatccttctcagctcctatctgtccagtgcaatgaacagca
NBPF10-1       gcagggatagctgagtcttcatcctcctcagctcctatctgttcagtgcactgaacacca
NBPF10-2       acagggatagctgagtcttcatcctcctcagctcctatctgtccagtgcaatgaacagca
NBPF11-1       acagggatagctgagtcttcatcctcttcagctcctatctgtccagtgcactgaacacca
NBPF11-2       acagggatagctgagtcttcatcctcctcagctcctatctgtccagtgcaatgaacagca
NBPF19         acagggatagctgagtcttcatcctcctcagctcctatctgtccagtgcaatgaacagca
NBPF26-1       acagggatagctgagtcttcatcctcctcagctcctatctgtccagtgcactgaacacca
NBPF26-2       acagggatagctgagtcttcatcctcctcagctcctatctgtccagtgcaatgaacagca
NBPF8          acagggacagctgagtcttcctcctcctcagctcctatctgcccagtgcaatgatcacta
NBPF4          acagggacagctgagtcttcctcctcctcagctcctatctgcccagtgcaatgatcacta
NBPF6          acagggacagctgagtcttcctcctcctcagctcctatctgcccagtgcaatgatcacta
                ** *** ******** *** ****  ** ******** **  ******* *** **  *
```

```
NBPF3         gttgctgtcttcctctctggctcccatggctgccatgctctgttgcagagagaagaggat
NBPF1-1       gctgctctcttcctctctggctcccatggcagccatgctctgttgcagagagaagaggat
NBPF1-2       gctgctctcttcctctctggctcccatggcagccatgctctgttgcagagagaagaggat
NBPF15        gctgctctcttcctctctggttcccatggcagccatgctctgttgcagagagaacaggat
NBPF14-1      gctgctctcttcctctctggctcccatggcagccatggtctgttgcagagagaagaggat
NBPF14-2      gctgctctcttcctctctggttcccatggcagccatgctctgttgcagagagaacaggat
NBPF20        gctgctctcttcctctctggttcccatggcagccatgctctgttgcagagagaacaggat
NBPF9-1       gctgctctcttcctctctggctcccatggcagccatggtctgttgcagagagaagaggat
NBPF9-2       gctgctctcttcctctctggttcccatggcagccatggtctgttgcagagagaagaggat
NBPF12        gctgctctcttcctctctggctcccatggcagccatgctctgttgcagagagaagaggat
NBPF12-1      gctgctctcttcctctctggctcccatggcagccatgctctgttgcagagagaagaggat
NBPF12-2      gctgctctcttcctctctggttcccatggcagccgtgctctgttgcagacagaacaggat
NBPF10-1      gctgctctcttcctctctggctcccatggcagccatggtctgttgcagagagaagaggat
NBPF10-2      gctgctctcttcctctctggttcccatggcagccatgctctgttgcagagagaacaggat
NBPF11-1      gctgctctcttcctctctggctcccatggcagcgaagctctgttgcagagagaagaggat
NBPF11-2      gctgctctcttcctctctggttcccatggcagccatgctctgttgcagacagaacaggat
NBPF19        gctgctctcttcctctctggttcccatggcagccatgctctgttgcagagagaacaggat
NBPF26-1      gctgctctcttcctctctggctcccatggcagccatgctctgttgcagagagaagcggat
NBPF26-2      gctgctctcttcctctctggttcccatggcagccatgctctgttgcagagagaacaggat
NBPF8         gctgctgtcttcctctctggttcccatggcagccacgctctgttgcagacagaaaaggat
NBPF4         gctgctgtcttcctctctggttcccatggcagccacgctctgttgcagacagaaaaggat
NBPF6         gctgctgtcttcctctctggttcccatggcagccacgctctgttgcagacagaaaaggat
              * **** ************* ********* **   * *********** ****  ****

NBPF3         tgcctgttccctcttaaagggatcctcctgtttgctttcttggaccactctcttaatgcc
NBPF1-1       tgcctgttccccccttaaagggaacctccattttgctttctgggaccactgtcttaatgcc
NBPF1-2       tgcctgttccccccttaaagggaacctccattttgctttctgggaccactgtcttaatgcc
NBPF15        tgcatgttccctcttaatgggaacgtccattttgctttctgggaccactctcttaatgcc
NBPF14-1      tgcctgttccctctttaagggaacctccgttttgctttctggaaccactctcttaatgcc
NBPF14-2      tgcatgttccctcttaatgggaacctccattttgctttctgggaccactctcttaatgcc
NBPF20        tgcatgttccccccttaatgggaacctccattttgctttctgggaccactctcttaatgcc
NBPF9-1       tgcctgttccctctttaagggaacctccgttttgctttctggaaccactctcttaatgcc
NBPF9-2       tacctgttccctcttaatgggaacctccattttgctttctgggaccactctcttaatgcc
NBPF12        tgcctgttccctctttaagggaacctccattttgctttctgggaccactctcttaatgcc
NBPF12-1      tgcctgttccctcttaaagggaacctccattttgctttctgggaccactctcttaatgcc
NBPF12-2      tgcatgttccctcttaatgggaacgtccattttgctttctgggaccactctcttaatgcc
NBPF10-1      tgcctgttccctctttaagggaacctccgttttgctttctggaaccactctcttaatgcc
NBPF10-2      tgcatgttccctcttaatgggaacctccattttgctttctgggaccactctcttaatgcc
NBPF11-1      tgcctgttccctcttaaagggaacctccatt-tgttttctgggaccactctcttaatgcc
NBPF11-2      tgcatgttccctcttaatgggaacgtccattttgctttctgggaccactctcttaatgcc
NBPF19        tgcatgttccctcttaatgggaacctccattttgctttctgggaccactctcttaatgcc
NBPF26-1      tgcctgtttcctctttaagggaacctccgttttgctttctggaaccactctcttaatgcc
NBPF26-2      tgcatgttccctcttaatgggaacctccattttgctttctgggaccactctcttaatgcc
NBPF8         tgcctgttccctcttaaaaggaacctctcctttgcattctgggaccactctcttaa-gcc
NBPF4         tgcctgttccctcttaaaaggaacctctcctttgcattctgggaccactctcttaa-gcc
NBPF6         tgcctgttccctcttaaaaggaacctctcctttgcattctgggaccactctcttaa-gcc
              * * **** ** *** *  *** * **   * **  **** * ****** ****** ***

NBPF3         tcctgtcgaaaccagctagcactccctggtgtccaatccctctgtgtttagtcttctgtc
NBPF1-1       gcctgtcaaaaccagctaggactccctggggtccaatccctctgtgtttaatcttctgtc
NBPF1-2       gcctgtcaaaaccagctaggactccctggggtccaatccctctgtgtttaatcttctgtc
NBPF15        gcctgtcaaaaccagctaggactccctggggtccaatccctctgtgtttaatcttctgtc
NBPF14-1      acctgtcaaaaccagctaggactccctggggtccaatccctctgtgtttaatcttctgtc
NBPF14-2      gcctgtcaaaaccagctaggactccctggggtccaatccctctgtgtttaatcttctgtc
NBPF20        gcctgtcaaaaccagctaggactccctggggtccaatccctctgtgtttaatcttctgtc
NBPF9-1       gcctgtcaaaaccagctagtactccctggggtccaatccctctgtgtttaatcttctgtc
NBPF9-2       gcctgtcaaaaccagctaggactccctggggtccaatccctctgtgtttaatcttctgtc
NBPF12        gcctgtcaaaaccagctaggactccctggggtccaatccctctgtgtttaatcttctgtc
NBPF12-1      gcttgtcaaaaccagctaggactccttggggtccaatccctctgtgtttaatcttctgtc
NBPF12-2      gcctgtcaaaaccagctaggactccctggggtccaatccctctgtgtttaatcttctgtc
NBPF10-1      gcctgtcaaaaccagctaggactccctggggtccaatccctctgtgtttaatcttctgtc
NBPF10-2      gcctgtcaaaaccagctaggactccctggggtccaatccctctgtgtttaatcttctgtc
NBPF11-1      gcttgtcaaaaccagctaggactccttggggtccaatccctctgtgtttaatcttctgtc
NBPF11-2      gcctgtcaaaaccagctaggactccctggggtccaatccctctgtgtttaatcttctgtc
NBPF19        gcctgtcaaaaccagctaggactccctggggtccaatccctctgtgtttaatcttctgtc
NBPF26-1      gcctgtcaaaaccagctaggactccatggggtccaatccctctgtgtttaatcttctgtc
NBPF26-2      gcctgtcaaaaccagctaggactccctggggtccaatccctctgtgtttaatcttctgtc
NBPF8         tcctttcaaaaccacctaggacttcttggggtgcaatgccttttggattaatcttctgtc
NBPF4         tcctttcaaaaccacctaggacttcttggggtgcaatgccttttggattaatcttctgtc
NBPF6         tcctttcaaaaccacctaggacttcttggggtgcaatgccttttggattaatcttctgtc
               * * ** ****** **** *** * *** ** **** *** *  * *** *********
```

```
                                         E  V  Q  K  A  E  E
NBPF3         atctctatcccacctggctcatcagGGAGGTGCAGAAGGCTGAAGAAAAGGAAGTCCCTG
NBPF1-1       atctctgtcccacctggctcatcagGGAGGTGCAGAAGGCTGAAGAAAGCAAAGTCCCTG
NBPF1-2       atctctgtcccacctggctcatcagGGAGGTGCAGAAGGCTGAAGAAAGCAAAGTCCCTG
NBPF15        atctctgtcccacctggctcatcagGGAGATGCAGAAGGCTGAAGAAAAGGAAGTCCCTG
NBPF14-1      atctctgtcccacctggctcatcagGGAGGTGCAGAAGGCTGAAGAGAGCAAAGTCCCTG
NBPF14-2      atctctgtcccacctggctcatcagGGAGATGCAGAAGGCTGAAGAAAAGGAAGTCCCTG
NBPF20        atctctgtcccacctggctcatcagGGAGATGCAGAAGGCTGAAGAAAAGGAAGTCCCTG
NBPF9-1       atctctgtcccacctggctcatcagGGAGGTGCAGAAGGCTGAAGAGAGCAAAGTCGCTG
NBPF9-2       atctctgtcccacctggctcatcagGGAGATGCAGAAGGCTGAAGAAAAGGAAGTCCCTG
NBPF12        atctctgtcccacctggctcatcagGGAGGTGCAGAAGGCTGAAGAGAGCAAAGTCCCTG
NBPF12-1      atctctgtcccacctggctcatcagGGAGATGCAGAAGGCTGAAGAAAGCAAAGTCCCTG
NBPF12-2      atctctgtcccacctggctcatcagGGAGATGCAGAAGGCTGAAGAAAAGGAAGTCCCTG
NBPF10-1      atctctgtcccacctggctcatcagGGAGGTGCAGAAGGCTGAAGAGAGCAAAGTCCCTG
NBPF10-2      atctctgtcccacctggctcatcagGGAGATGCAGAAGGCTGAAGAAAAGGAAGTCCCTG
NBPF11-1      atctctgtcccacctggctcatcagGGAGGTGCAGAAGGCTGAAGAGAGCAAAGTCCCTG
NBPF11-2      atctctgtcccacctggctcatcagGGAGATGCAGAAGGCTGAAGAAAAGGAAGTCCCTG
NBPF19        atctctgtcccacctggctcatcagGGAGATGCAGAAGGCTGAAGAAAAGGAAGTCCCTG
NBPF26-1      atctctgtcccacctggctcatcagGGAGGTGCAGAAGACTGAAGAGAGCAAAGTCCCTG
NBPF26-2      atctctgtcccacctggctcatcagGGAGATGCAGAAGGCTGAAGAAAAGGAAGTCCCTG
NBPF8         atctctatcccactgggctcatcagAGAGGTGCAGAAGACTGAAGAAAAGGAAGTCCCTC
NBPF4         atctctatcccactgggctcatcagAGAGGTGCAGAAGACTGAAGAAAAGGAAGTCCCTC
NBPF6         atctctatcccactgggctcatcagAGAGGTGCAGAAGACTGAAGAAAAGGAAGTCCCTC
              ****** ******  ********** *** ******** ******* *   ***** **

NBPF3         AGGACTCACTGGAGGAGTGTGCCATCACTTGTTCAAATAGCCACCACCCTTGTGAGTCCA
NBPF1-1       AGGACTCACTGGAGGAATGTGCCATCACTTGTTCAAATAGCCACAGCCCTTGTGACTCCA
NBPF1-2       AGGACTCACTGGAGGAATGTGCCATCACTTGTTCAAATAGCCACGGCCCTTGTGACTCCA
NBPF15        AGGACTCACTGGAGGAATGTGCCATCACTTGTTCAAATAGCCATGGCCCTTATGACTCCA
NBPF14-1      AGGACTCACTGGAGGAATGTGCCATCACTTGTTCAAATAGCCACGGCCCTTGTGACTCCA
NBPF14-2      AGGACTCACTGGAGGAATGTGCCATCACTTATTCAAATAGCCATGGCTCTTATGACTCCA
NBPF20        AGGACTCACAGGAGGAATGTGCCATCACTTATTCAAATAGCCATGGCCCTTATGACTCCA
NBPF9-1       AGGACTCACTGGAGGAATGTGCCATCACTTGTTCAAATAGCCACGGCCCTTGTGACTCCA
NBPF9-2       AGGACTCACTGGAGGAATGTGCCATCACTTATTCAAATAGCCATGGCCCTTATGACTCCA
NBPF12        AGGACTCACTGGAGGAATGTGCCATCACTTGTTCAAATAGCCACGGCCCTTGTGACTCCA
NBPF12-1      AGGACTCACTGGAGGAATGTGCCATCACTTGTTCAAATAGCCACGGCCCTTGTGACTCCA
NBPF12-2      AGGACTCACTGGAGGAGTGTGCCATCACTTGTTCAAATAGCCATGGCCCTTATGACTCCA
NBPF10-1      AGGACTCACTGGAGGAATGTGCCATCACTTGTTCAAATAGCCACGGCCCTTGTGACTCCA
NBPF10-2      AGGACTCACTGGAGGAATGTGCCATCACTTATTCAAATAGCCATGGCTCTTATGACTCCA
NBPF11-1      AGGACTCACTGGAGGAATGTGCCATCACTTGTTCAAATAGCCACGGCCCTTGTGACTCCA
NBPF11-2      AGGACTCACTGGAGGAGTGTGCCATCACTTGTTCAAATAGCCATGGCCCTTATGACTCCA
NBPF19        AGGACTCACTGGAGGAATGTGCCATCACTTATTCAAATAGCCATGGCCCTTATGACTCCA
NBPF26-1      AGGACTCACTGGAGGAATGTGCCATCACTTGTTCAAATAGCCACGGCCCTTGTGACTCCA
NBPF26-2      AGGACTCACTGGAGGAATGTGCCATCACTTGTTCAAATAGCCATGGCCCTTATGACTGCA
NBPF8         AGGACTCACTGGAGGAATGTGCTGTCACTTGTTCAAATAGTCACAACCCTTCTAACTCCA
NBPF4         AGGACTCACTGGAGGAATGTGCTGTCACTTGTTCAAATAGTCACAACCCTTCTAACTCCA
NBPF6         AGGACTCACTGGAGGAATGTGCTGTCACTTGTTCAAATAGTCACAACCCTTCTAACTCCA
              ********* ****** *****  ****** ********* **   * *** * * * **

NBPF3         ACCAGCCTTACGGGAACACCAGAATCACATTTGAGGAAGACCAAGTCGACTCAACTCTCA
NBPF1-1       ACCAGCCTCACAAGAACATCAACATCACCTTTGAGGAAGACAAAGTCAACTCAACTCTGG
NBPF1-2       ACCAGCCTCACAAGAACATCAACATCACCTTTGAGGAAGACAAAGTCAACTCAGCTCTGG
NBPF15        ACCAGCCACATAAGAAAACCAAAATCACATTTGAGGAAGACAAAGTCGACTCAACTCTCA
NBPF14-1      ACCAGCCTCACAAGAACATCAAAATCACATTTGAGGAAGACGAAGTCAACTCAACTCTGG
NBPF14-2      ACCAGCCACATAGGAAAACCAAAATCACATTTGAGGAAGACAAAGTCGACTCAACTCTCA
NBPF20        ACCAGCCACATAGGAAAACCAAAATCACATTTGAGGAAGACAAAGTCGACTCAACTCTGA
NBPF9-1       ACCAGCCTCACAAGAACATCAAAATCACATTTGAGGAAGACGAAGTCAACTCAACTCTGG
NBPF9-2       ACCAGCCACATAGGAAAACCAAAATCACATTTGAGGAAGACAAAGTCGACTCAACTCTCA
NBPF12        TCCAGCCTCACAAGAACATCAAAATCACATTTGAGGAAGACAAAGTCAACTCAACTGTGG
NBPF12-1      ACCAGCCTCACAAGAACATCAAAATCACATTTGAGGAAGACAAAGTCAACTCATCTCTGG
NBPF12-2      ACCAGCCACATAGGAAAACCAAAATCACATTTGAGGAAGACAAAGTCGACTCAACTCTCA
NBPF10-1      ACCAGCCTCACAAGAACATCAAAATCACATTTGAGGAAGACGAAGTCAACTCAACTCTGG
NBPF10-2      ACCAGCCACATAGGAAAACCAAAATCACATTTGAGGAAGACAAAGTCGACTCAACTCTCA
NBPF11-1      TCCAGCCTCACAAGAACATCAAAATCACATTTGAGGAAGACAAAGTCAACTCATCTCTGG
NBPF11-2      ACCAGCCACATAGAAAAACCAAAATCACATTTGAGGAAGACAAAGTCGACTCAACTCTCA
NBPF19        ACCAGCCACATAGGAAAACCAAAATCACATTTGAGGAAGACAAAGTCGACTCAACTCTCA
NBPF26-1      ACCAGCCTCACAAGAACATCAAAATCACATTTGAGGAAGACGAAGTCAACTCAACTCTGG
NBPF26-2      ACCAGCCACATAGGAAAACCAAAATCACATTTGAGGAAGACAAAGTCGACTCAACTCTCA
NBPF8         ACCAGCCTCACAGGAGCACCAAAATCACATTTAAGGAACACGAAGTCGACTCTGCTCTGG
NBPF4         ACCAGCCTCACAGGAGCACCAAAATCACATTTAAGGAACACGAAGTCGACTCTGCTCTGG
NBPF6         ACCAGCCTCACAGGAGCACCAAAATCACATTTAAGGAACACGAAGTCGACTCTGCTCTGG
               ******  *    *  * **  ***** *** ***** ** ***** ****  ** *
```

```
NBPF3         TTGA------CTCATCCTCTCATGATGAATGGTTGGATGCTGTATGCATTATCCCAGgtagcc
NBPF1-1       TTGTAGACAGAGAATCCTCTCATGATGAATGTCAGGATGCTGTAAACATTCTCCCAGgtagcc
NBPF1-2       TTGTAGACAGAGAATCCTCTCATGATGAATGTCAGGATGCTGTAAACATTCTCCCAGgtagcc
NBPF15        TTGG------CTCATCCTCTCATGTTGAATGGGAGGATGCTGTACACATTATTCCAGgtagcc
NBPF14-1      TTGTAGACAGAGAATCCTCTCATGATGAATGTCAGGATGCTCTAAACATTCTCCCAGgtagcc
NBPF14-2      TTGG------CTCATCCTCTCATGTTGAATGGGAGGATGCTGTACACATTATTCCAGgtagcc
NBPF20        TTGG------CTCATCCTCTCATGTTGAATGGGAGGATGCTGTACACATTATTCCAGgtagcc
NBPF9-1       TTGTAGACAGAGAATCCTCTCATGATGAATGTCAGGATGCTCTAAACATTCTCCCAGgtagcc
NBPF9-2       TTGG------CTCATCCTCTCATGTTGAACGGGAAGATGCTGTACACATTATTCCAGgtagcc
NBPF12        TTGTAGACAGAAAATCCTCTCATGATGAATGTCAGGATGCTCTAAACATTCTCCCAGgtagcc
NBPF12-1      TTGTAGACAGAGAATCCTCTCATGATGAATGTCAGGATGCTCTAAACATTCTCCCAGgtagcc
NBPF12-2      TTGG------CTCATCCTCTCATGTTGAATGGGAGGATGCTGTACACATTATCCCAGgtagcc
NBPF10-1      TTGTAGACAGAGAATCCTCTCATGATGAATGTCAGGATGCTCTAAACATTCTCCCAGgtagcc
NBPF10-2      TTGG------CTCATCCTCTCATGTTGAATGGGAGGATGCTGTACACATTATTCCAGgtagcc
NBPF11-1      TTGTAGACAGAGAATCCTCTCATGATGGATGTCAGGATGCTCTAAACATTCTCCCAGgtagcc
NBPF11-2      TTGG------CTCATCCTCTCATGTTGAATGGGAGGATGCTGTACACATTATCCCAGgtagcc
NBPF19        TTGG------CTCATCCTCTCATGTTGAATGGGAGGATGCTGTACACATTATTCCAGgtagcc
NBPF26-1      TTGTAGACAGAGAATCCTCTCATGATGAATGTCAGGATGCTCTAAACATTCTCCCAGgtagcc
NBPF26-2      TTGG------CTCATCCTCTCATGTTGAATGGGAGGATGCTGTACACATTATTCCAGgtagcc
NBPF8         TTGTAGAGAGTGAACACCCTCATGATGAAGAGGAGGAAGCTCTAAACATTCCCCCAGgtagcc
NBPF4         TTGTAGAGAGTGAACACCCTCATGATGAAGAGGAGGAAGCTCTAAACATTCCCCCAGgtagcc
NBPF6         TTGTAGAGAGTGAACACCCTCATGATGAAGAGGAGGAAGCTCTAAACATTCCCCCAGgtagcc
              ***          *  * ****** ** *      ** *** **  ****   **********
```

SUPPLEMENTAL TABLES

Table S1 Synthetic nucleic acids

**Oligo(ribo)nucleotide**	**5'-3' sequence[1]**	**Reference**
**siRNAs**		
SRSF1	ACGAUUGCCGCAUCUACGU	(6)
SRSF1	CCAAGGACAUUGAGGACGU	(7,8)
SRSF2	CAAGCAACUGGCUAUUGAA	(9)
SRSF2	AAUCCAGGUCGCGAUCGAA	(9,10)
SRSF3	GGAAAUAGAAGACAGUUUG	(9,11)
SRSF3	GGUCCCUUUCUAGAGAUAG	(9,11)
SRSF3	GAGUGGAACUGUCGAAUGG	(12)
SRSF3	CGAAGUGUGUGGGUUGCUA	(12)
SRSF4	AGACCAAGCUGAAGAGAAG	(9)
SRSF4	AGCAGUCAUUCUAAGAGUA	(9)
SRSF5	UGCUCCACCUGUAAGAACA	(9)
SRSF5	ACGUGGUUCUUCAAGUAGA	(9)
SRSF6	GCAGAUCUAAGGAUGAGUA	(9)
SRSF6	AAGAUGAGGCUCUAAGGAA	(9)
SRSF7	AGAUCAAGAUCCAGGUCUA	(13)
SRSF9	AGAGGAUGCUAUUUAUGGA	(14)
Tra2α	GCAUGAAGACUUUCUGAAA	(9)
Tra2β	AGGUAGUGUAAUCGCCUUG	(9)
**Cloning primers**		
SRSF1-F	ACCAGGATCCTTTTCGTCACCGCCATGT	This study
SRSF1-R	ACCACTCGAGATCATCATATGTACGAGAGCGAGA	This study
SRSF2-F	ACCAGGATCCGGCCGCCACTCAGAGCTA	This study
SRSF2-R	ACCACTCGAGATAAGAGGACACCGCTCCTTC	This study
SRSF5-F	ACCAAAGCTTGACATCATGAGTGGCTGTCG	This study
SRSF5-R	ACCACTCGAGATTGCCACTGTCAACTGATCTGG	This study
SRSF6-F	ACCAGGATCCTTCGACAACCAGCCCTTG	This study
SRSF6-R	ACCACTCGAGATCTCTGGAACTCGACCTGGA	This study
**RT-PCR**		
35E1+PL4	CAGGTGCTCTCGGTTGCA	(15)
35m-amplF	GCTCGGATCCTACAGAGTCAA	(15)
PL4	AGTCGAGGCTGATCAGCGG	(9)
**Mutagenic inactivation of exon 4a**		
3'ss	TTATTGCTTCCGGGTCAGGGGTC	This study
5'ss	TTCAAACGTGGATGAGAATTAAGC	This study
**Mutagenic inactivation of exon 4b**		
3'ss	TTGGCCACTCATGGATACGAGGGCA	This study
5'ss	CAGACAAACTTGATGAGGGTCTGAG	This study
**DADLD mutagenesis**		
HADLD	CTGGGGATTTTG<u>CACGCTGATCTGGAC</u>T	(15)
DAHLD	GGATTTTG<u>GATGCTCACCTGGAC</u>TCCTCC	(15)
DADLH	G<u>GATGCTGATCTGCAC</u>TCCTCCGTGC	(15)

[1]Mutated residues are in red. Nucleotides coding for the DADLD motif are underlined.

Table S2 Candidate RBPs for promoting inclusion of exons that encode CBSs

Protein	Domain	Preferred GAN trimer within optimal hexa-/nona-mers	Amino acid	Reference
ANKHD1	KH	GAC	Asp	(16)
CNOT4	RRM	GAC	Asp	(16)
ENOX1	RRM	GAC	Asp	(16)
FXR1	KH	GAC	Asp	(16)
FXR2	KH	GAC	Asp	(16)
RBM45	RRM	GAC	Asp	(16)
SRSF7	RRM, zinc finger	GAC	Asp	(17)
G3BP2	RRM	GAU	Asp	(16)
HNRNPH2	RRM	GAG	Glu	(16)
LIN28A	Cold shock domain, zinc finger	GAG	Glu	(18)
PABPC1	RRM	GAA	Glu	(16)
PABPC5	RRM	GAA	Glu	(16)
PABPN1	RRM	GAA	Glu	(16)
RBM5	Zinc finger	GAA	Glu	(16)
SART3	RRM	GAA	Glu	(16)
SRSF1	RRM	GAG	Glu	(19)
SRSF2	RRM	GAG	Glu	(17)
SRSF9	RRM	GAG	Glu	(14)
SRSF9	RRM	GAA	Glu	(16)
SRSF10	RRM	GAG	Glu	(19)
TARDBP	RRM	GAA	Glu	(16)
Tra2α	RRM	GAA	Glu	(20,21)
Tra2β	RRM	GAA	Glu	(20)

Table S3 Nucleotide composition of exonic sequences encoding canonical EF-hand loops

	%A	%C	%T	%G
**Mean**	31.62	18.75	17.62	32.01
**SD**	7.68	7.70	6.82	7.02

Table S4 Intrinsic strength of SR-activated cryptic splice sites in *OGDH* transcripts

OGDH exon	Splice site	Splice-site sequence	Maximum entropy score
4a	Cryptic 3'ss-44	tcttttaaccctccccacagCTC	10.51
4a	3'ss	gcttgtgttattgcttccagGTC	8.85
4a	5'ss	TGGgtgaga	6.04
4a	Cryptic 5'ss+32	AATgtaatt	1.50
4b	Cryptic 3'ss+7	tggccactcatagatacgagGGC	-16.18
4b	3'ss	tcatcgttggccactcatagATA	3.36
4b	5'ss	TTGgtgagg	6.90
4b	Cryptic 5'ss+134	TCGgtattg	3.83

Table S5 Key residues for binding common divalent metals are encoded by both fragile and robust codons

I-W binding affinity	Metal	Protein binding residues[1]	Codons[1]
Weak	Ca	Asp, Glu	GAU, GAC, GAA, GAG
Weak	Mg	Asp, Glu	GAU, GAC, GAA, GAG
Intermediate	Mn	Asp, Glu, His	GAU, GAC, GAA, GAG, CAU, CAC,
Intermediate	Fe	Cys, His, Glu,	UGU, UGC, CAU, CAC, GAA, GAG
Strong	Cu	His, Cys	CAU, CAC, UGU, UGC
Strong	Zn	His, Cys, Glu	CAU, CAC, UGU, UGC

[1]Fragile (underlined) and robust residues and codons were defined by Cusack et al. (4).

Table S6 m^6A sites reported in EF-hand loop codons

Genomic coordinates of m^6A[1]	Canonical EF-hand[2]	EF-hand loop codon with m^6A
chr15:78105804-78105805	*CIB2_3*	GACTTG**GAC**GGTGACGGCAAGCTGGGCTTTGCTGAC
chr11:64854459-64854460	*EHD1_1*	GACGTGGACAAG**GAC**GGGCTGCTGGACGACGAGGAG
chr19:47741286-47741287	*EHD2_1*	GATGTG**GAC**CGCGACGGCATGCTGGATGATGAGGAG
chr2:46907842-46907843	*MCFD2_1*	GATTATGATGGCAATAATTTGCTTGATGGCTTA**GAA**
chr19:48919209-48919210	*NUCB1_2*	GACACCAACCAG**GAC**CGCCTCGTGACCCTGGAGGAG
chr2:46905493-46905494	*MCFD2_2*	GACAAGAACAATGATGGATACATT**GAC**TATGCTGAA
chr9:83062770-83062771	*RASEF_1*	GACGCGAACCGCTCGGGGCGCCTGGAGCGCGAGGAG[3]
chr19:11445460-11445461	*PRKCSH_1*	GATGAT**GAC**ATGGACGGGACGGTCTCGGTGACTGAG

[1] Human genome reference GRCh38; [2]Gene symbol is followed by the EF-hand number, as in Dataset S1. The overlap was obtained using a total of 36,556 m^6A sites (accession number GSE163500) predicted by m6Aboost (22). [3]m^6A is adjacent to codon 12 of the first EF-hand loop.

Table S7 Occurrence of preferred m^{6}A targets in exonic sequences for EF-hand motifs and Ca^{2+}-binding loops

m^{6}A pentamer/ trimer targets	EF-hand loops (10,656 nt)	EF-hand motifs (31,968 nt)	Control exons (34,247,622 nt)	Fold enrichment (loop *vs* controls)	Fold enrichment (loop *vs* motif)	Fold enrichment (motif *vs* controls)
GGACU	14	32	67524	0.67	1.31	0.51
GAACU	9	59	72420	0.40	0.46	0.87
AGACU	6	24	59612	0.32	0.75	0.43
GGAUU	11	32	52412	0.67	1.03	0.65
GGACG	28	81	41618	2.16	1.04	2.09
GAC triplets	494	793	880795	1.80	1.87	0.96

REFERENCES TO SUPPLEMENTAL INFORMATION

1. Gingold, H., Tehler, D., Christoffersen, N.R., Nielsen, M.M., Asmar, F., Kooistra, S.M., Christophersen, N.S., Christensen, L.L., Borre, M., Sorensen, K.D. *et al.* (2014) A dual program for translation regulation in cellular proliferation and differentiation. *Cell*, **158**, 1281–1292.
2. Yeo, G. and Burge, C.B. (2004) Maximum entropy modeling of short sequence motifs with applications to RNA splicing signals. *J. Comput. Biol.*, **11**, 377-394.
3. Ke, S., Shang, S., Kalachikov, S.M., Morozova, I., Yu, L., Russo, J.J., Ju, J. and Chasin, L.A. (2011) Quantitative evaluation of all hexamers as exonic splicing elements. *Genome Res.*, **21**, 1360–1374.
4. Cusack, B.P., Arndt, P.F., Duret, L. and Crollius, H.R. (2011) Preventing dangerous nonsense: selection for robustness to transcriptional error in human genes. *PLoS Genet.*, **7**, e1002276.
5. Popesco, M.C., MacLaren, E.J., Hopkins, J., Dumas, L., Cox, M., Meltesen, L., McGavran, L., Wyckoff, G.J. and Sikela, J.M. (2006) Human lineage-specific amplification, selection, and neuronal expression of DUF1220 domains. *Science*, **313**, 1304-1307.
6. Karni, R., de Stanchina, E., Lowe, S.W., Sinha, R., Mu, D. and Krainer, A.R. (2007) The gene encoding the splicing factor SF2/ASF is a proto-oncogene. *Nat. Struct. Mol. Biol.*, **14**, 185-193.
7. Blaustein, M., Pelisch, F., Tanos, T., Munoz, M.J., Wengier, D., Quadrana, L., Sanford, J.R., Muschietti, J.P., Kornblihtt, A.R., Caceres, J.F. *et al.* (2005) Concerted regulation of nuclear and cytoplasmic activities of SR proteins by AKT. *Nat. Struct. Mol. Biol.*, **12**, 1037-1044.
8. Raponi, M., Kralovicova, J., Copson, E., Divina, P., Eccles, D., Johnson, P.M., Baralle, D. and Vorechovsky, I. (2011) Prediction of single-nucleotide substitutions that result in exon skipping: identification of a splicing silencer in *BRCA1* exon 5. *Hum. Mutat.*, **32**, 436-444.
9. Kralovicova, J. and Vorechovsky, I. (2010) Allele-dependent recognition of the 3' splice site of *INS* intron 1. *Hum. Genet.*, **128**, 383-400.
10. Gabut, M., Mine, M., Marsac, C., Brivet, M., Tazi, J. and Soret, J. (2005) The SR protein SC35 is responsible for aberrant splicing of the E1alpha pyruvate dehydrogenase mRNA in a case of mental retardation with lactic acidosis. *Mol. Cell. Biol.*, **25**, 3286-3294.
11. Bedard, K.M., Daijogo, S. and Semler, B.L. (2007) A nucleo-cytoplasmic SR protein functions in viral IRES-mediated translation initiation. *EMBO J.*, **26**, 459-467.
12. Kim, J., Park, R.Y., Chen, J.K., Kim, J., Jeong, S. and Ohn, T. (2014) Splicing factor SRSF3 represses the translation of programmed cell death 4 mRNA by associating with the 5'-UTR region. *Cell Death Differ.*, **21**, 481-490.
13. Gao, L., Wang, J., Wang, Y. and Andreadis, A. (2007) SR protein 9G8 modulates splicing of tau exon 10 via its proximal downstream intron, a clustering region for frontotemporal dementia mutations. *Mol. Cell. Neurosci.*, **34**, 48-58.

14. Paradis, C., Cloutier, P., Shkreta, L., Toutant, J., Klarskov, K. and Chabot, B. (2007) hnRNP I/PTB can antagonize the splicing repressor activity of SRp30c. *RNA*, **13**, 1287-1300.
15. Kralovicova, J., Borovska, I., Pengelly, R., Lee, E., Abaffy, P., Sindelka, R., Grutzner, F. and Vorechovsky, I. (2021) Restriction of an intron size *en route* to endothermy. *Nucleic Acids Res.*, **49**, 2460-2487.
16. Ray, D., Kazan, H., Cook, K.B., Weirauch, M.T., Najafabadi, H.S., Li, X., Gueroussov, S., Albu, M., Zheng, H., Yang, A. *et al.* (2013) A compendium of RNA-binding motifs for decoding gene regulation. *Nature*, **499**, 172-177.
17. Cavaloc, Y., Bourgeois, C.F., Kister, L. and Stevenin, J. (1999) The splicing factors 9G8 and SRp20 transactivate splicing through different and specific enhancers. *RNA*, **5**, 468-483.
18. Lehrbach, N.J., Armisen, J., Lightfoot, H.L., Murfitt, K.J., Bugaut, A., Balasubramanian, S. and Miska, E.S. (2009) LIN-28 and the poly(U) polymerase PUP-2 regulate *let-7* microRNA processing in *Caenorhabditis elegans*. *Nat. Struct. Mol. Biol.*, **16**, 1016-1020.
19. Ray, D., Kazan, H., Chan, E.T., Pena Castillo, L., Chaudhry, S., Talukder, S., Blencowe, B.J., Morris, Q. and Hughes, T.R. (2009) Rapid and systematic analysis of the RNA recognition specificities of RNA-binding proteins. *Nat. Biotechnol.*, **27**, 667-670.
20. Tsuda, K., Someya, T., Kuwasako, K., Takahashi, M., He, F., Unzai, S., Inoue, M., Harada, T., Watanabe, S., Terada, T. *et al.* (2010) Structural basis for the dual RNA-recognition modes of human Tra2β RRM. *Nucleic Acids Res.*, **39**, 1538-1553.
21. Grellscheid, S., Dalgliesh, C., Storbeck, M., Best, A., Liu, Y., Jakubik, M., Mende, Y., Ehrmann, I., Curk, T., Rossbach, K. *et al.* (2011) Identification of evolutionarily conserved exons as regulated targets for the splicing activator tra2beta in development. *PLoS Genet.*, **7**, e1002390.
22. Kortel, N., Ruckle, C., Zhou, Y., Busch, A., Hoch-Kraft, P., Sutandy, F.X.R., Haase, J., Pradhan, M., Musheev, M., Ostareck, D. *et al.* (2021) Deep and accurate detection of m6A RNA modifications using miCLIP2 and m6Aboost machine learning. *Nucleic Acids Res.*, **49**, e92.
